# Origin and evolution of plexins, semaphorins, and Met receptor tyrosine kinases

**DOI:** 10.1038/s41598-019-38512-y

**Published:** 2019-02-13

**Authors:** Chrystian Junqueira Alves, Karla Yotoko, Hongyan Zou, Roland H. Friedel

**Affiliations:** 1 Fishberg Department of Neuroscience, Friedman Brain Institute, Icahn School of Medicine at Mount Sinai, New York, New York, 10029 USA; 2 Department of Neurosurgery, Friedman Brain Institute, Icahn School of Medicine at Mount Sinai, New York, New York, 10029 USA; 3 Biology Department, Universidade Federal de Viçosa, Viçosa, Minas Gerais 36570-900 Brazil

**Keywords:** Molecular evolution, Evolutionary biology

## Abstract

The transition from unicellular to multicellular organisms poses the question as to when genes that regulate cell-cell interactions emerged during evolution. The receptor and ligand pairing of plexins and semaphorins regulates cellular interactions in a wide range of developmental and physiological contexts. We surveyed here genomes of unicellular eukaryotes and of non-bilaterian and bilaterian Metazoa and performed phylogenetic analyses to gain insight into the evolution of plexin and semaphorin families. Remarkably, we detected plexins and semaphorins in unicellular choanoflagellates, indicating their evolutionary origin in a common ancestor of Choanoflagellida and Metazoa. The plexin domain structure is conserved throughout all clades; in contrast, semaphorins are structurally diverse. Choanoflagellate semaphorins are transmembrane proteins with multiple fibronectin type III domains following the N-terminal Sema domain (termed Sema-FN). Other previously not yet described semaphorin classes include semaphorins of Ctenophora with tandem immunoglobulin domains (Sema-IG) and secreted semaphorins of Echinoderamata (Sema-SP, Sema-SI). Our study also identified Met receptor tyrosine kinases (RTKs), which carry a truncated plexin extracellular domain, in several bilaterian clades, indicating evolutionary origin in a common ancestor of Bilateria. In addition, a novel type of Met-like RTK with a complete plexin extracellular domain was detected in Lophotrochozoa and Echinodermata (termed Met-LP RTK). Our findings are consistent with an ancient function of plexins and semaphorins in regulating cytoskeletal dynamics and cell adhesion that predates their role as axon guidance molecules.

## Introduction

Plexins and semaphorins are cell surface receptors and ligands that regulate multiple processes of development and adult physiology, including neural development, vascular development, immune system activation, bone homeostasis, and epithelial organization^1–9^.

Semaphorins were identified as repulsive axon guidance molecules in grasshopper and in chick^10,11^. Plexins were first described as cell surface molecules in the nervous system of Xenopus tadpoles^12^ and as molecules with sequence similarity to the extracellular domain of Met receptor tyrosine kinases (RTKs)^13^.

Semaphorins of bilaterian Metazoa have been grouped by sequence similarity into two invertebrate classes (class 1 and 2) and five vertebrate classes (class 3–7), plus one class for viral semaphorins (class V)^14,15^. Subsequent studies revealed that *Drosophila melanogaster* also has a class 5 semaphorin, thus class 5 semaphorins are present in both vertebrates and invertebrates^16^. The viral semaphorins appear to be all derived from class 7 semaphorins^17^. The plexins of vertebrates were grouped by sequence similarity into 4 classes (class A-D)^18^.

Both plexins and semaphorins contain a structural hallmark: an N-terminal Sema domain, which is a seven-blade beta-propeller, with each blade formed by four anti-parallel beta-strands^19^. The Sema domain is exclusive of plexins, semaphorins, and Met RTKs^19^. Plexins and semaphorins bind to each other through their respective Sema domains^20–22^. Met RTKs, on the other hand, bind to secreted growth factors such as hepatocyte growth factor (HGF) that are structurally different from semaphorins^23^.

For the binding of secreted class 3 semaphorins, neuropilin transmembrane proteins act as required co-receptors in a complex with plexins^24^. To date, Neuropilins are considered exclusive of vertebrates^25^, although in *Caenorhabditis elegans*, the L1CAM homolog LAD-2 acts as co-receptor for secreted semaphorins^26^, suggesting that invertebrates may utilize other co-receptors for the binding of secreted semaphorins.

The Sema domain of plexins is typically followed by a PSI domain (plexins, semaphorins, and integrins), which is ~50–60 aa in size and contains up to 8 conserved cysteines with a central conserved CxWC motif ^27^. Plexins also contain extracellular IPT domains (immunoglobin-like fold shared by plexins and transcription factors), which are ~95 aa in size, forming a beta-sheet bundle with two central conserved cysteines^27^. It has been an intriguing finding from crystallographic studies that the plexin extracellular domains form together a ring structure, which consists of a Sema domain followed by three consecutive PSI-IPT domains and three more IPT domains, i.e. Sema-PSI-IPT-PSI-IPT-PSI-IPT-IPT-IPT-IPT-TM^28,29^. Exceptions to this stereotypic domain pattern are Plexin-B1, which carries an insert in its second PSI domain, and Plexin-C1, which lacks one PSI and two IPT domains.

The intracellular part of plexins contains a Ras-GAP (GTPase activating protein) domain, which inactivates small G proteins of the Rap1/2 families^30^. The Rap1/2 G proteins have a pleiotropic network of effectors that control cytoskeletal dynamics and cell adhesion processes^31^.

Previous genome sequencing studies have recognized that plexins and semaphorins have an early origin in metazoan evolution^32,33^. In the current study, we surveyed plexins and semaphorins from unicellular eukaryotes to complex metazoan organisms to track their evolutionary origin and to describe their intra- and extracellular domains arrangements. Remarkably, we detected plexins and semaphorins in choanoflagellates, a group of unicellular eukaryotes closely related to Metazoa^34^. Our phylogenetic analyses suggest that plexins and semaphorins emerged in a common ancestor of choanoflagellates and Metazoa. Whereas plexins have maintained a stable domain arrangement throughout all clades, semaphorins have evolved into diverse classes with different domain arrangements. Interestingly, our analyses of Sema domain-containing proteins also revealed a novel type of Met-like RTK that contains a complete plexin ectodomain structure.

## Results

### Survey for Sema domain-containing proteins in genome databases

We compiled predicted protein sequences of Sema domain-containing proteins (plexins, semaphorins, and Met RTKs) from genome databases of unicellular eukaryotes and Metazoa by keyword searches (“plexin”, “sema”, and “semaphorin”) and BLAST searches (see Methods). Protein domains were annotated using conserved domain and secondary structure prediction algorithms (see Methods).

Protein name assignment was guided by our phylogenetic analysis based on Sema plus PSI domain sequences, protein domain arrangements, and naming conventions for related proteins in *C*. *elegans*, *D*. *melanogaster*, and *H*. *sapiens*^14,18^. Plexins of each species were numbered starting with the suffix -1 for the molecule that branches most basally from the phylogenetic tree. Plexins forming monophyletic groups within a species (*i*.*e*. that duplicated within a species) were numbered starting with the suffix -A1. For semaphorins, we followed the bilaterian naming conventions, except for the semaphorins that were distinct in domain arrangement from previously described classes and were therefore assigned new class names with numerical index. The protein names of this study should be understood as preliminary effort, and future committee work will be required to revise the nomenclature.

### Plexins and semaphorins are present in unicellular choanoflagellates

To assess if plexins and semaphorins are present in unicellular eukaryotes, we first examined species of the clades Ichtyosporea, Filasterea, and Choanoflagellida, which are phylogenetically closely related to Metazoa^34,35^. No Sema domain-containing proteins were detected in the genomes of the ichtyosporean *Creolimax fragrantissima*^36^ or the filasterean *Capsaspora owczarzaki*^37^. In contrast, one plexin and one semaphorin each were found in the genomes of the choanoflagellates *Monosiga brevicollis* and *Salpingoeca rosetta*^38,39^.

**Figure 1:**
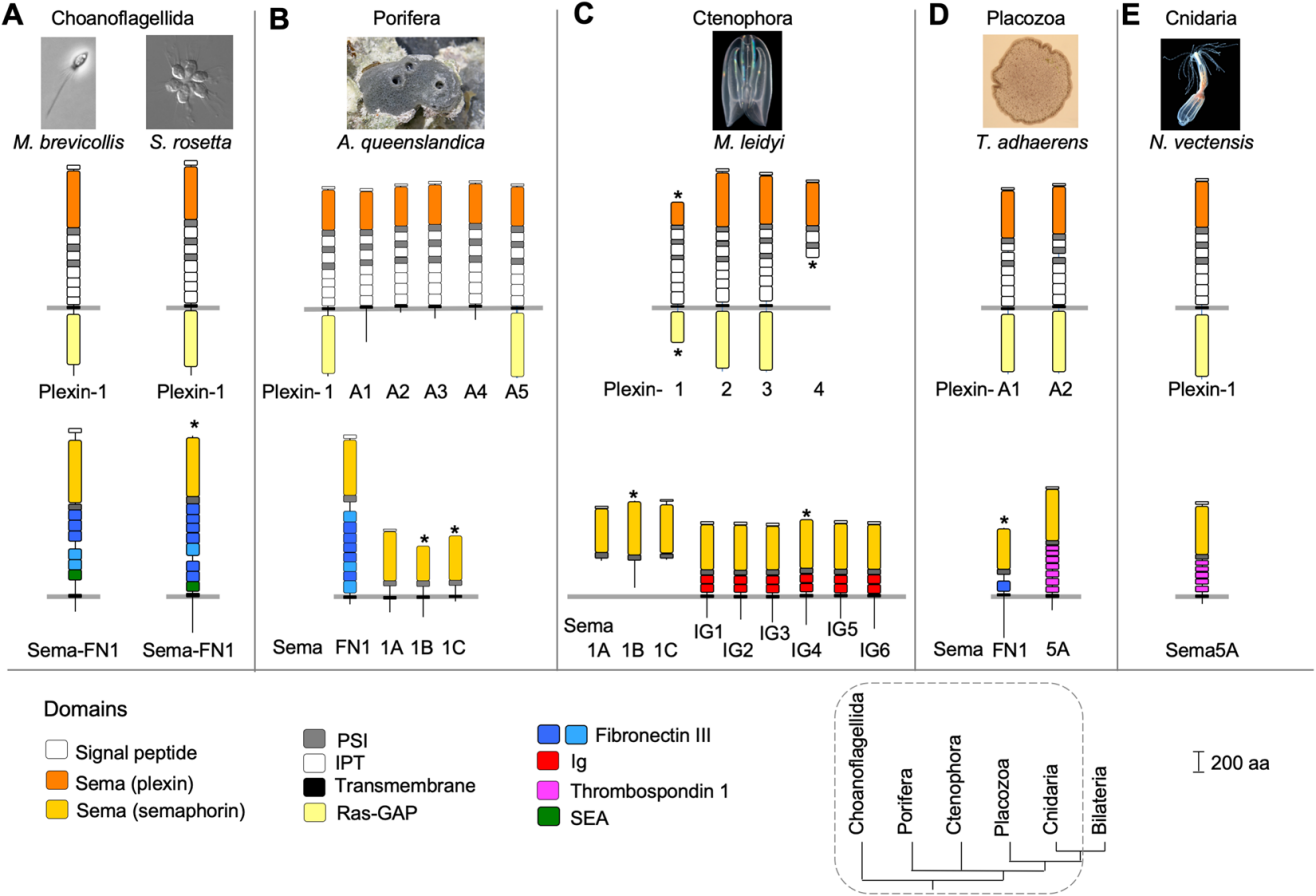
Plexins and semaphorins of choanoflagellates and non-bilaterian Metazoa. (**A**) The genomes of choanoflagellates *M*. *brevicollis and S*. *rosetta* each contain one plexin (Plexin-1) and one semaphorin with fibronectin type III (FNIII) and SEA domains (Sema-FN1). (**B**) The sponge *A*. *queenslandica* has six plexins, four of which with truncated intracellular domain. The four transmembrane semaphorins have a Sema plus PSI architecture (Sema1A-1C) or carry in addition fibronectin type III domains (Sema-FN1). (**C**) The comb jelly *M*. *leidyi* has four plexins, and two classes of semaphorins: Sema1A-1C are secreted semaphorins with a Sema plus PSI architecture, Sema-IG1 to -IG6 are transmembrane semaphorins with two Ig domains in tandem arrangement. (**D**) The placozoan *T*. *adhaerens* has two plexins, Plexin-A1 and -A2, and two transmembrane semaphorins: Sema-FN1 with a single FNIII domain, and Sema5A with the typical architecture of class 5 semaphorins containing multiple thrombospondin 1 (TSP1) domains. (**E**) The cnidarian *N*. *vectensis* has one Plexin-1 and one Sema5A with multiple TSP1 domains. Protein domains: PSI, domain found in plexins, semaphorins, integrins; IPT, immunoglobulin-like fold shared by plexins and transcription factors; Ras-GAP, Ras GTPase activating protein; Ig: immunoglobulin domain. Darker blue for FNIII domains indicates higher annotation certainty. Asterisks indicate missing sequence information. Phylogenetic tree after^35^; dashed line encircles the clades shown in this figure. Photo credits: *M*. *brevicollis* and *S*. *rosetta:* Mark Dayel^64^ (mark@dayel.com; http://www.dayel.com/choanoflagellates; CC BY-SA 3.0; https://creativecommons.org/licenses/by-sa/3.0/legalcode); *A*. *queenslandica*: Marcin Adamski; *M*. *leidyi:* William Browne; *T*. *adhaerens*: Oliver Voigt; *N*. *vectensis*: Eric Roettinger.

The choanoflagellate plexins, which we termed Plexin-1 (Fig. 1A), are remarkably similar in their domain structure to the plexins of Metazoa and contain an extracellular N-terminal Sema domain, followed by three consecutive PSI-IPT domains and three additional IPT domains, and then an intracellular Ras-GAP domain (Fig. 1A). The semaphorins of choanoflagellates are transmembrane proteins that contain an N-terminal Sema plus PSI domain, followed by an extracellular stalk with five to seven fibronectin type III (FNIII) domains and a SEA domain (Fig. 1A). This domain architecture could reflect one of the earliest forms of semaphorins, which we termed Sema-FN, reflecting the presence of *FN*III domains. No conserved intracellular domains were detected in Sema-FN1.

### Conservation of plexin structure from choanoflagellates to humans

**Figure 2:**
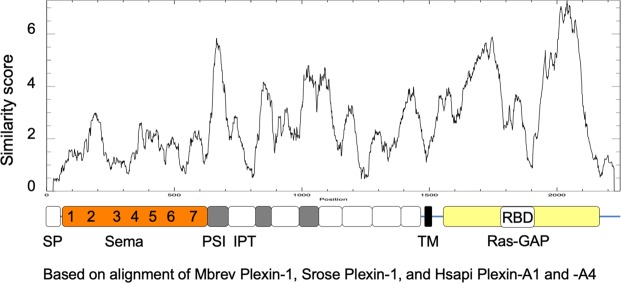
Conservation between choanoflagellate and human plexins. Plot of protein sequence conservation between choanoflagellate Plexin-1 (*S*. *rosetta* and *M*. *brevicollis*) and human Plexin-A1 and Plexin-A4. Overall domain arrangement is conserved, with the highest degree of sequence conservation in the Ras-GAP domain, interrupted by the insert of a lesser conserved RBD domain. The conservation pattern of the Sema domain reflects the seven blade propeller structure. Window size for similarity scoring was set to 50 aa. The y-axis indicates similarity score as calculated by plotcon algorithm (http://emboss.open-bio.org/rel/dev/apps/plotcon.html). See also Figs S1–S3 for alignments and secondary structure prediction.

To further examine the similarity between choanoflagellate and human plexins, we aligned the sequences of Plexin-1 of *M*. *brevicollis and S*. *rosetta* with those of human plexins and generated a conservation plot (Fig. 2; see Fig. S1 for alignment). The best conserved part is the Ras-GAP domain, which may reflect the importance of catalytic Ras-GAP activity for plexin signaling^30^. In contrast, the insert of the Rho-binding domain (RBD), which is thought to have a regulatory function^40^, is less conserved, suggesting that regulation of plexin activity may involve different Rho family members in diverse ways. The conservation pattern of the Sema domain indicated a seven blade propeller pattern. Likewise, protein secondary structure prediction (JPRED) of the choanoflagellate Plexin-1 Sema domain (Fig. S2) revealed a pattern of beta-strands that is typical of the seven propeller blades of Sema domains^19^. Interestingly, the conservation of the PSI and IPT domains was overall higher than that of the Sema domain, possibly reflecting an important role for the extracellular domains in forming a ring topology, a specific aspect of plexin structure^28,29^.

Alignments of Sema domains from plexins and semaphorins of choanoflagellates and humans revealed up to eight conserved cysteine residues (Fig. S3). Jansen *et al*. (2010)^20^ and Nogi *et al*. (2010)^22^ have proposed that such residues maintain the structural integrity of the Sema domain in humans. In contrast, residues that are part of known binding surfaces of plexins and semaphorins were not highly conserved, perhaps reflecting the diversity of plexin/semaphorin receptor/ligand pairings (Fig. S3).

### Survey of non-bilaterian Metazoa: Plexins and multiple semaphorin classes

We next searched for plexins and semaphorins in the genomes of Porifera (sponges), Ctenophora (comb jellies), Placozoa, and Cnidaria, four non-bilaterian lineages that form the sister group of the remaining Metazoa^34,35^. Of note, the order of the phylogenetic branches of Porifera and Ctenophora is still a matter of debate^32,41,42^.

In the genome of the sponge *Amphimedon queenslandica*^43^, we detected two full-length plexins with conserved domain structure, termed Plexin-1 and Plexin-A5 (Fig. 1B), and four plexins that contain only relatively short intracellular parts with no conserved domains, termed Plexin-A1 to -A4. We also found four transmembrane semaphorins in *A*. *queenslandica*: Sema-FN1, which contains a Sema plus PSI domain and several FNIII repeats (five FNIII domains detected by sequence homology; but based on spacing and cysteine patterns, up to nine FNIII domains may be present), as well as Sema-1A to -1C, which contain a Sema plus PSI domain.

Unlike sponges, comb jellies have a developed nervous system. We found in the genome of the ctenophore *Mnemiopsis leidyi*^32^ four plexins, termed Plexin-1 to -4 (Fig. 1C). We also detected nine semaphorins: three secreted semaphorins, Sema-1A to -1C, each with a Sema plus PSI domain, and six transmembrane semaphorins, Sema-IG1 to -IG6, each with a Sema plus PSI domain followed by two tandem immunoglobulin (*Ig*) domains (Fig. 1C).

*Trichoplax adhaerens* is an animal with a simple two-cell layer structure and the only known extant member of the clade Placozoa. We found in the *T*. *adhaerens* genome^44^ two plexins, Plexin-A1 and -A2 (Fig. 1D). The predicted protein sequences of these plexins appear to contain small deletions between their first and third PSI-IPT domains (however, it is presently unclear if these deletions reflect gaps in genomic coverage). We also detected in *T*. *adhaerens* two transmembrane semaphorins: Sema-FN1 has a Sema plus PSI domain and a single FNIII domain; Sema5A is structurally related to the Sema5s of bilaterian Metazoa and contains an array of seven thrombospondin type-1 (TSP1) domains following the Sema plus PSI domain.

In the genome of the sea anemone *Nematostella vectensis*, a member of the taxon Cnidaria^45^, we detected a single Plexin-1 and a single transmembrane semaphorin with five detectable TSP1 domains, termed Sema5A (Fig. 1E).

### Survey of Protostomia: Plexins, semaphorins, and novel Met-LP RTKs

Protostomia and Deuterostomia are the two main clades of bilaterian Metazoa. We first analyzed Protostomia by surveying the genomes of the annelid *Capitella teleta*^46^, the mollusk *Aplysia californica*^47^, and the chilopod *Strigamia maritima*^48^. We also included for reference the well-characterized plexins and semaphorins of *C*. *elegans* (wormbase.org) and *D*. *melanogaster* (flybase.org).

**Figure 3:**
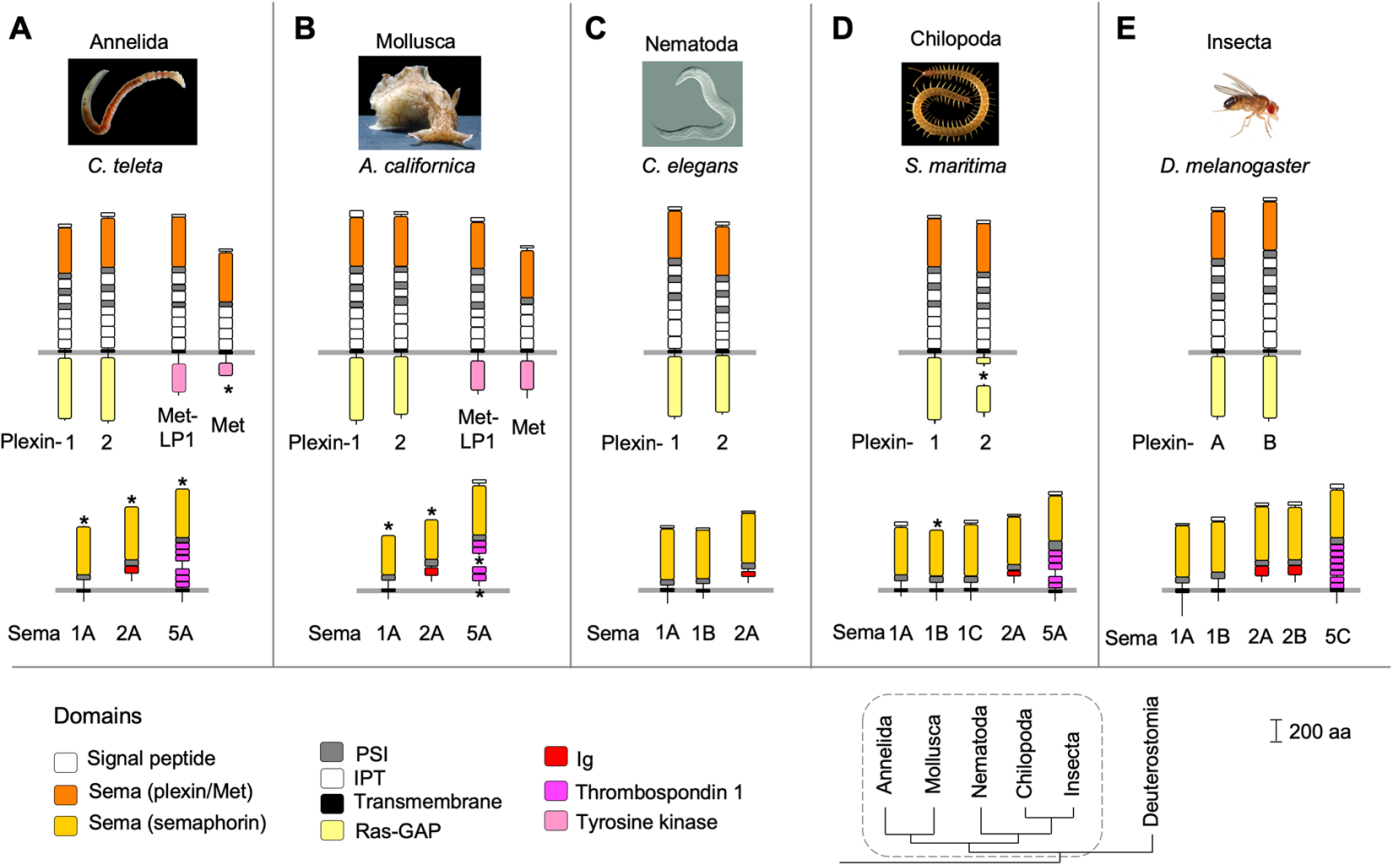
Plexins, semaphorins, and Met and Met-LP RTKs of Protostomia. (**A**,**B**) The genomes of the annelid *C*. *teleta* (**A**) and the sea hare *A*. *californica* (**B**) each contain two plexins: Plexin-1 and -2, and three semaphorins: Sema1A, 2A, and 5A. One Met and one Met-LP RTK each were also detected. (**C**) The nematod *C*. *elegans* has two plexins and three semaphorins (wormbase.org). (**D**) The centipede *S*. *maritima* has two plexins: Plexin-1 and -2, and five semaphorins: Sema1A-1C, 2A, and 5A. (**E**) The fruit fly *D*. *melanogaster* has two plexins and five semaphorins (flybase.org). Protein domains: see legend of Fig. 1. Asterisks indicate missing sequence. Dashed line in phylogenetic tree encircles clades shown in this figure. Photo credits: *C*. *teleta*: François Michonneau (https://francoismichonneau.net/photos/capitella-telata) © 2018 by François Michonneau (CC-BY 4.0); *A*. *californica:* Genevieve Anderson*; C*. *elegans:* Bob Goldstein; *S*. *maritima*: Carlo Brena*; D*. *melanogaster*: Hans Smid.

In both *C*. *teleta* and *A*. *californica*, we detected two Plexins, termed Plexin-1 and -2 (Fig. 3A,B). We also detected in each species three semaphorins that are structurally similar to the Sema1, 2, and 5 classes of *C*. *elegans* and *D*. *melanogaster* (Fig. 3C,E), termed accordingly Sema1A, Sema2A, and Sema5A. In *S*. *maritima*, we detected two Plexins, termed Plexin-1 and -2, three transmembrane semaphorins with the Sema plus PSI domain, termed Sema1A to 1 C, one secreted Sema2A, and one transmembrane Sema5A (Fig. 3D).

We detected in both annelid and mollusk genomes Met RTKs, with a domain structure like human Mets, consisting of a truncated plexin-like extracellular domain with the Sema plus PSI domain, followed by four IPT domains, and an intracellular tyrosine kinase domain (Fig. 3A,B). This finding was unexpected as Met RTKs had so far been assumed to be absent from Protostomia^49^. Interestingly, we also detected in Annelida and Mollusca a novel type of Met-like RTK, termed Met-*l*ike with *p*lexin ectodomain (Met-LP), which contains an extracellular domain with a complete plexin ectodomain structure and an intracellular tyrosine kinase domain (Fig. 3A,B).

### Survey of Deuterostomia: Expansion of plexins and semaphorins in Echinodermata and Vertebrata

To survey Deuterostomia, we analyzed the genomes of the starfish *Acanthaster planci*^50^ and the lancelet *Branchiostoma belcheri*^51^. We also added for reference human plexins, semaphorins, and Met RTKs (genenames.org) to represent the clade Vertebrata.

**Figure 4:**
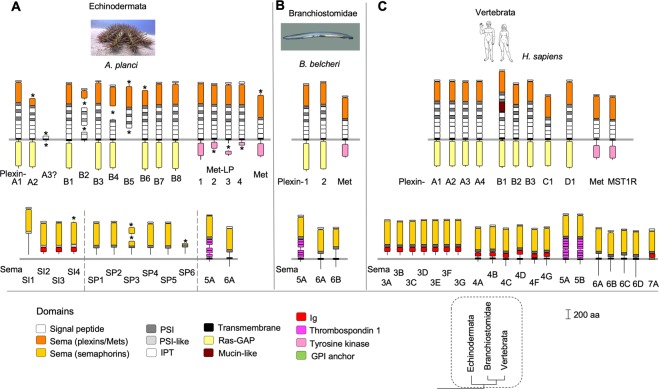
Plexins, semaphorins, Met, and Met-LP RTKs of Deuterostomia. (**A**) Expansion of plexins and semaphorins occurred in the genome of the starfish *A*. *planci*. Eleven plexins were detected, grouped into two classes (Plexin-A1 to -A3 and Plexin-B1 to -B8), as well and four Met-LP and one Met RTK. Two classes of secreted semaphorins were detected: Sema-SI with a Sema domain, a PSI-like domain, and an Ig domain (Sema-SI1 to -SI4), and Sema-SP with a Sema plus PSI domain. Additionally, the transmembrane semaphorins Sema5A and Sema6A are detected. (**B**) The lancelet *B*. *belcheri* has two plexins, one Met RTK, and three transmembrane semaphorins: Sema5A, 6A, and 6B. (**C**) Expansion of plexins and semaphorins occurred in the lineage of Vertebrata, as exemplified by the nine plexins, twenty semaphorins, and two Met RTKs in the genome of *H*. *sapiens* (genenames.org). Protein domains: see legend of Fig. 1. Asterisks indicate missing sequence. Dashed line in phylogenetic tree encircles the clades shown in this figure. Photo/image credits: *A*. *planci*: Denis Zorzin; *B*. *belcheri*: Arthur Anker; *H*. *sapiens:* NASA.

In the genome of *A*. *planci*, we detected a substantial expansion of the plexin, semaphorin, and Met-LP families (Fig. 4A). Interestingly, an expansion of plexins and semaphorins had also been previously reported for the sea urchin *Strongylocentrotus purpuratus*^52^. For instance, we detected in *A*. *planci* two Plexin-A molecules, Plexin-A1 and -A2, and also a short fragment of a potential third Plexin-A3. We found eight plexins in *A*. *planci* that formed a phylogenetically distinct group from the Plexin-As (see phylogeny below), termed Plexin-B1 to -B8. In addition, one Met receptor tyrosine kinase was found, along with four Met-LPs. The semaphorins of *A*. *planci* fall into four classes: one semaphorin is a member of class 5, termed Sema5A; one a member of class 6, termed Sema6A; four secreted semaphorins that carry after the *S*ema domain an atypical PSI-like domain (lacking some conserved cysteines) and an *I*g domain, termed Sema-SI1 to -SI4 (of note, Sema-SI1 lacks the Ig domain); and six secreted semaphorins that carry a *S*ema plus *P*SI domain, termed Sema-SP1 to -SP6.

We detected in the genome of the lancelet *B*. *belcheri* a compact set of plexins and semaphorins, with two plexins, Plexin-1 and -2, and three semaphorins, Sema5A, 6A, and 6B, and also one Met (Fig. 4B).

### Evolution of Sema domain-containing proteins

To gain further insight into the evolutionary relationship of plexins and semaphorins, as well as Met and Met-LP RTKs, we inferred a phylogenetic hypothesis based on the Sema plus PSI domains of all the proteins described in this study.

**Figure 5:**
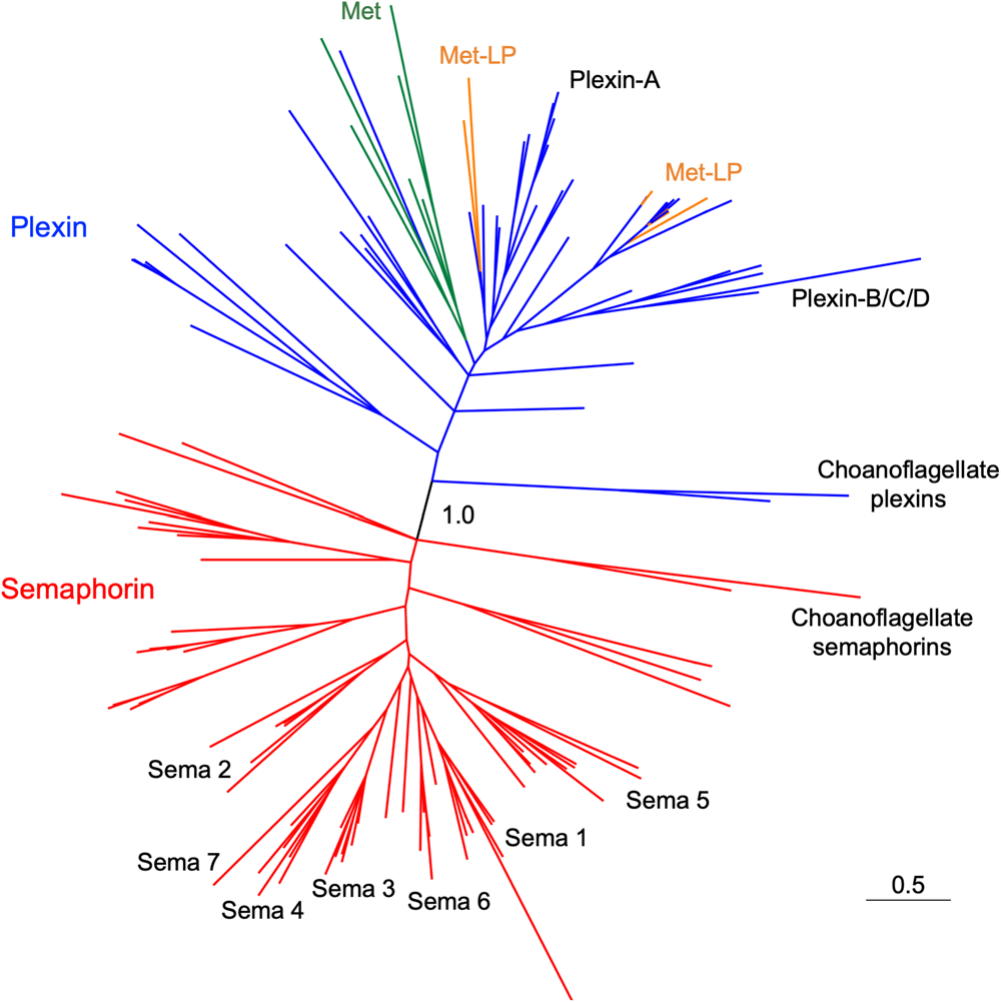
Phylogenetic tree of Sema domain containing proteins. Unrooted representation of a phylogenetic hypothesis inferred by a Bayesian method with the aid of the software MrBayes 3.2.6 implemented on CIPRES^63^ based on alignment of all Sema plus PSI domain sequences of all proteins described in this study. The “1.0” label in the center is the posterior probability at this branch point calculated by MrBayes. Red lines indicate semaphorins, blue lines plexins, green lines Met RTKs, and orange lines Met-LP RTKs. The plexins and semaphorins of choanoflagellates branch off from the base of the respective plexin and semaphorin clades. Human plexin classes (Plexin-A and Plexin-B/C/D) and bilaterian semaphorin classes (Sema1–7) are indicated to provide reference to the detailed tree representations shown in Figs 6 and 7. Scale bar represents peptide sequence divergence of the sequences.

In an unrooted overview representation of the tree (Fig. 5), semaphorins form a monophyletic clade including the Choanoflagellida and Metazoa sequences (in red), separated from another monophyletic clade comprising plexins of Choanoflagellida and Metazoa, Mets, and Met-LPs. This topology indicates that choanoflagellates and Metazoa inherited at least one plexin and one semaphorin from a common ancestor. Met RTKs appear as a monophyletic group inside the plexin clade, while Met-LP RTKs are a polyphyletic group (see below).

**Figure 6:**
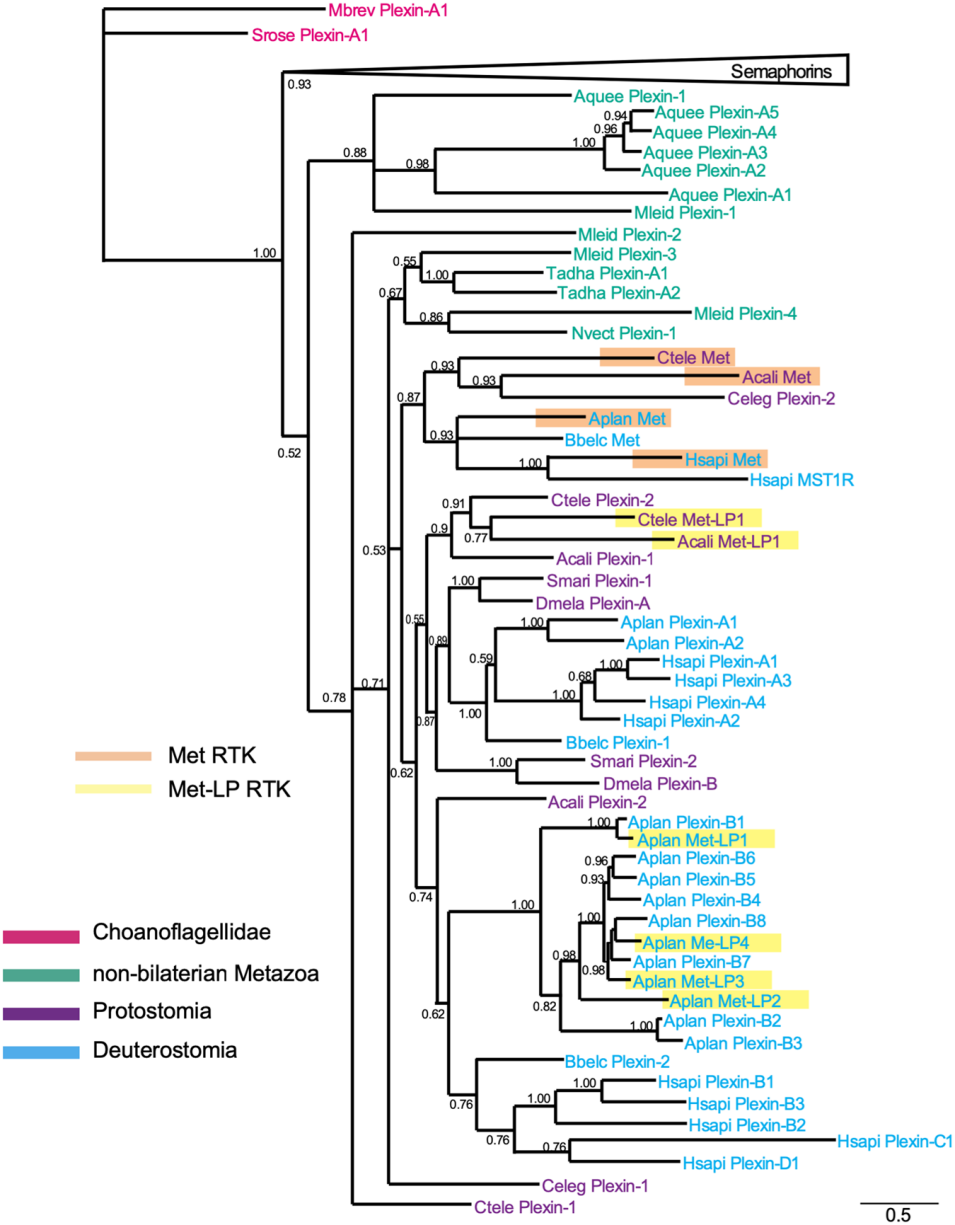
Phylogenetic tree of plexins and Met and Met-LP RTKs. Detailed phylogenetic hypothesis (complete hypothesis shown in Fig. 5) of plexin, Met, and Met-LP sequences. For clarity, semaphorin sequences are collapsed (triangle). Numbers beside each internal node correspond to posterior probability of the Bayesian phylogenetic inference of each group calculated by MrBayes 3.2.6. Met-LPs and Mets are highlighted by shaded orange and yellow boxes, respectively. The branch lengths are proportional to scale bar and represent the peptide sequence divergence of the sequences included in the tree. Species acronyms: Choanoflagellida (in red): Mbrev, *M*. *brevicollis;* Srose, *S*. *rosetta;* non-bilaterian Metazoa (in green): Aquee, *A*. *queenslandica;* Mleid, *M*. *leidyi;* Tadha, *T*. *adhaerens;* Nvect, *N*. *vectensis;* Protostomia (in purple): Ctele, *C*. *teleta*; Acali, *A*. *californica;* Celeg, *C*. *elegans*; Smari, *S*. *maritima;* Dmela, *D*. *melanogaster;* Deuterostomia (in blue): Aplan, *A*. *planci*; Bbelc, *B*. *belcheri*; Hsapi, *H*. *sapiens*.

**Figure 7:**
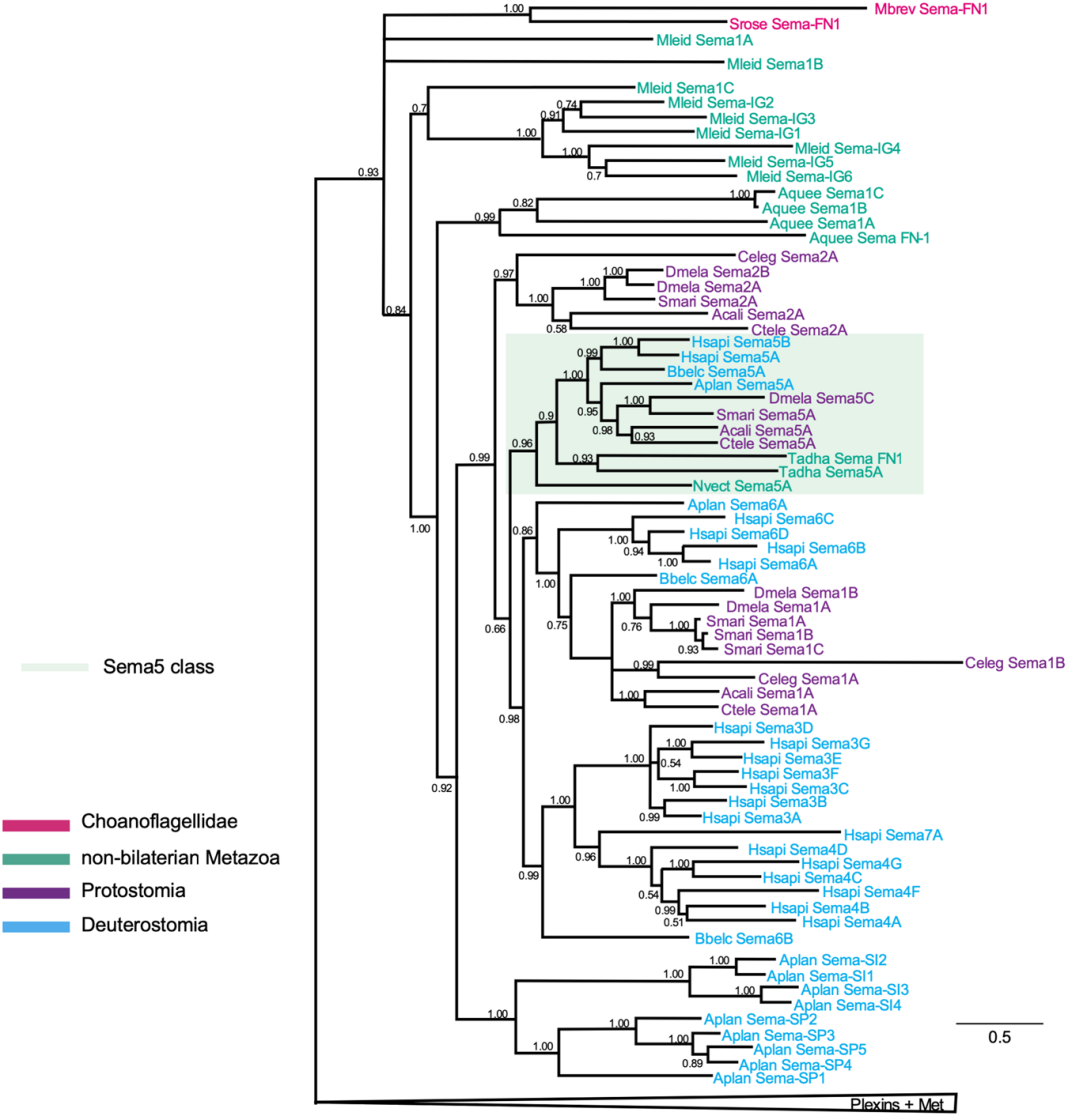
Phylogenetic tree of semaphorins. Detailed phylogenetic hypothesis (complete hypothesis shown in Fig. 5) of semaphorin sequences. For clarity, plexin, Met, and Met-LP sequences are collapsed (triangle). Numbers next to internal nodes correspond to posterior probability of the Bayesian phylogenetic inference of each group calculated by MrBayes 3.2.6. The orthologous group of class 5 semaphorins is highlighted by shaded green box. The branch lengths are proportional to scale bar and represent peptide sequence divergence of the sequences included in the tree. Species acronyms: Choanoflagellida (in red): Mbrev, *M*. *brevicollis;* Srose, *S*. *rosetta;* non-bilaterian Metazoa (in green): Aquee, *A*. *queenslandica;* Mleid, *M*. *leidyi;* Tadha, *T*. *adhaerens;* Nvect, *N*. *vectensis;* Protostomia (in purple): Ctele, *C*. *teleta*; Acali, *A*. *californica;* Celeg, *C*. *elegans*; Smari, *S*. *maritima;* Dmela, *D*. *melanogaster;* Deuterostomia (in blue) Aplan, *A*. *planci*; Bbelc, *B*. *belcheri*; Hsapi, *H*. *sapiens*.

We next visualized the phylogeny of plexins (with Mets and Met-LPs) and semaphorins in detailed trees that included Bayesian posterior probabilities (Fig. 6 and Fig. 7). The tree topologies suggest that genes of ancestral plexins/semaphorins repeatedly duplicated during metazoan evolution. We also inferred a minimal number of gene duplication events that can explain the current number of copies of plexins and semaphorins in each genome by mapping hypothetical ancestral genes onto the best phylogenetic hypothesis proposed for the taxa^34^ (see Figs S4,S5).

Following the topology of the phylogenetic hypothesis inferred for plexins (Fig. 6), we observed that after the split of Choanoflagellida sequences, the tree contains the collapsed semaphorins (posterior probability (PP) = 0.93) and all Metazoa plexins (PP = 0.52). Inside the Metazoa plexin clade, we found as a sister group (PP = 0.88) a clade containing all Porifera (*A*. *queenslandica;* Aquee) plexins and Ctenophora (*M*. *leidyi;* Mleid) Plexin-1. The next step of the tree showed a polytomic clade (PP = 0.78), containing as clades a Ctenophora sequence (Mleid Plexin-2), an Annelida protein (*C*. *teleta*; Ctele Plexin-1), and a major third polytomic clade (PP = 0.71) that contains all other plexin sequences.

The first branch of this polytomy is a plexin of Nematoda (*C*. *elegans*; Celeg Plexin-1), the second branch (PP = 0.67) contains the remaining non-bilaterian plexins, with proteins of Placozoa (*T*. *adhaerens*; Tadha Plexin-A1 and A2), Ctenophora (Mleid Plexin-4), and Cnidaria (*N*. *vectensis*; Nvect Plexin-1), and the third branch (PP = 0.53) containing only Protostomia and Deuterostomia sequences.

The first branch (PP = 0.87) of this Protostomia/Deuterstomia-only clade contains all Met sequences, and also Celeg Plexin-2. The second branch (PP = 0.62) splits again into two major branches. One major branch (PP = 0.55) contains a clade with Protostomia Ctele Plexin-2 and Acali Plexin-1, and the Met-LPs of these taxa; and a clade with plexins of Insecta and Chilopoda (*D*. *melanogaster*; Dmela Plexin-B and *S*. *maritima*; Smari Plexin-2), and a clade that branches into one clade with Smari Plexin-1 and Dmela Plexin-A and another one with all Deuterostomia Plexin-1/Plexin-A proteins (*H*. *sapiens*; Hsapi Plexin-A1-A4; *A*. *planci*; Aplan Plexin-A1/A2; *B*. *belcheri*; Bbelc Plexin-1). The second major branch (PP = 0.74) has the Mollusca Acali Plexin-2 as a sister species of a clade (PP = 0.62) divided in two: the first one comprising Echinodermata Aplan Plexin-Bs and Aplan Met-LPs (PP = 1.0), and the second comprising Branchiostomidae Bbelc Plexin-2 and human Plexins-Bs, Plexin-C1, and -D1 (PP = 0.76).

The topology of the phylogenetic tree of semaphorins (Fig. 7) shows that such sequences start with a basal polytomy of four branches. The first (PP = 1.0) contains the Choanoflagellidae semaphorins (in red), the second and the third consist of Ctenophora sequences (Mleid Sema-1A and 1B), and the fourth contains all the remaining sequences (PP = 0.84). This group splits again into two clades, the first one (PP = 0.7) contains Mleid Sema1C and a monophyletic group (PP = 1.00) comprising all the Mleid semaphorins with an IG domain.

The second clade (PP = 1.0) splits into two major branches, of which the first clade (PP = 0.99) contains all the Porifera Aquee sequences of this tree. The second clade (PP = 0.92) is divided into two other clades, the first contains only Echinodermata sequences (PP = 1.0) and splits into two other clades, the first (PP = 1.0) containing the Aplan Sema-SI class and the second (PP = 1.0) the Aplan Sema-SP class. The second clade (PP = 0.99) is divided into two clades, the first (PP = 0.97) contains the Protostomia Sema2s while the other comprises the remaining semaphorins (PP = 0.66). This clade is divided into two further clades, the first (PP = 0.96) comprises the Sema5 class, in which all members contain a series of thrombospondin domains. The second clade (PP = 0.98) splits into two clades: the first (PP = 0.86) with semaphorins of Protostomia (Sema1 class) and Deuterostomia (Sema6 class); and the second (PP = 0.99) with Bbelc Sema6B plus the Hsapi Sema3, 4, and 7 sequences.

**Figure 8:**
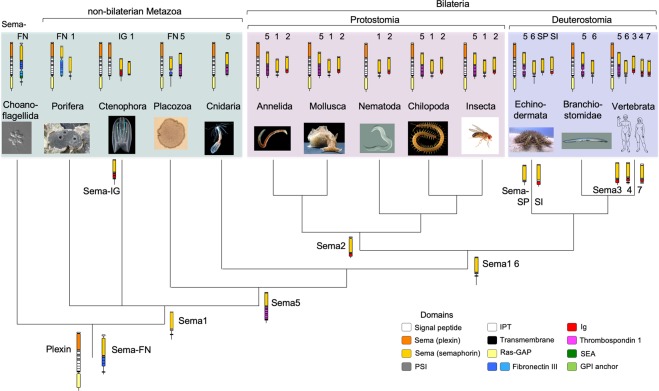
Model of evolutionary origin of plexins and semaphorins. Protein models above taxon names indicate presence of plexin and semaphorin classes in the respective clade (semaphorin class names denoted). Potential origins of semaphorin classes in ancestral lineages are indicated. A plexin and a class FN semaphorin may have been present in a common ancestor of Chonaflagellida and Metazoa. Loss of FN domains may have led to class 1 semaphorins of non-bilaterian Metazoa. The Sema5 class may have originated in a common ancestor of Placozoa, Cnidaria, and Bilateria (note absence in Nematoda). Semaphorins with Ig domains may have evolved twice independently, in Ctenophora as Sema-IG, and in Bilateria as classes Sema2, -SI, 3, 4, and 4. Protein domains: see legends of Figs 1, 3, and 4. Photo/image credits: *M*. *brevicollis* and *S*. *rosetta:* Mark Dayel^64^ (mark@dayel.com; http://www.dayel.com/choanoflagellates; CC BY-SA 3.0; https://creativecommons.org/licenses/by-sa/3.0/legalcode); *A*. *queenslandica*: Marcin Adamski; *M*. *leidyi:* William Browne; *T*. *adhaerens*: Oliver Voigt; *N*. *vectensis*: Eric Roettinger; *C*. *teleta*: François Michonneau (https://francoismichonneau.net/photos/capitella-telata) © 2018 by François Michonneau (CC-BY 4.0); *A*. *californica:* Genevieve Anderson*; C*. *elegans:* Bob Goldstein; *S*. *maritima*: Carlo Brena*; D*. *melanogaster*: Hans Smid; *A*. *planci*: Denis Zorzin; *B*. *belcheri*: Arthur Anker; *H*. *sapiens:* NASA.

The phylogenetic and structural data led us to a preliminary model about the evolutionary origin of plexins and the different semaphorin classes (Fig. 8). Plexins and semaphorins may have evolved in a common ancestor of choanoflagellates and Metazoa. The complete plexin domain structure may have already been present in this ancestor, and remained largely constant during metazoan evolution. One of the first ancestral semaphorins may have been of the transmembrane Sema-FN class, containing a Sema plus PSI domain followed by fibronectin domains (gene 1 in Fig. S5A). Losses of FN domains may have occurred in lineages of several non-bilaterian Metazoa, leading to class 1 semaphorins of non-bilaterian Metazoa. A class 5 semaphorin (gene 7 in Fig. S5A) with thrombospondin domains may have originated in a common ancestor of Placozoa and Metazoa (of note, class 5 semaphorins are absent from Nematoda, likely due to gene loss). The Sema-IG class is likely to have evolved in the Ctenophora lineage, and Sema-SP and -SI classes the Echinodermata lineage. Interestingly, the gain of an Ig domain appears to have occurred twice independently in semaphorin evolution, in the lineages leading to Ctenophora (Sema-IG) and Bilateria. Thus, in Bilateria, the Sema2 class of Protostomia and the Sema-SI, 3, 4, and 7 classes of Deuterostomia may have evolved from an ancestral semaphorin gene that gained an Ig domain in the lineage leading to Bilateria (gene 1-IG in Fig. S5A).

### Evolution of Met and Met-LP RTKs

**Figure 9:**
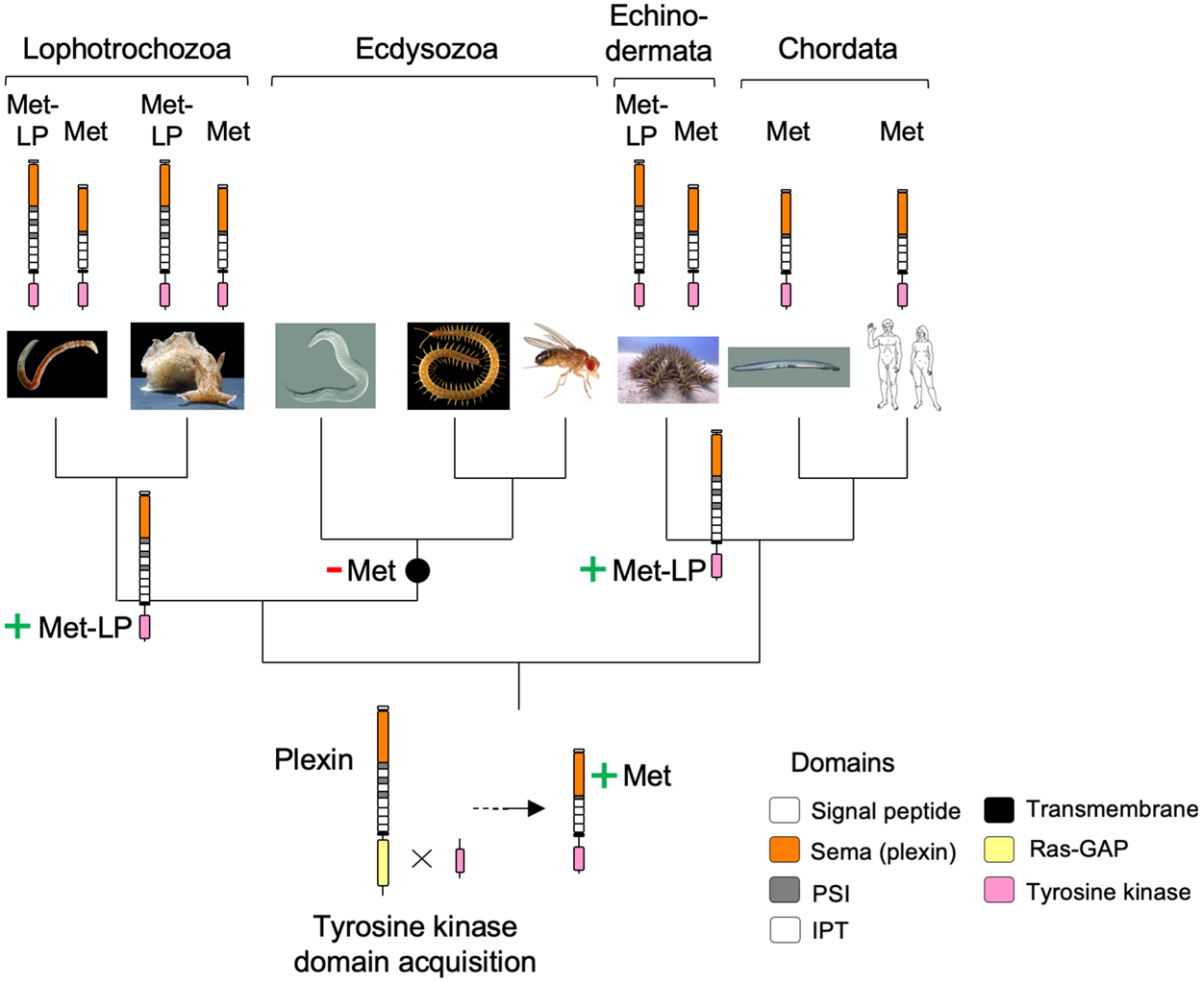
Model of evolutionary origin of Met and Met-LP RTKs. Protein models below taxon brackets indicate presence of Met-LPs and/or Mets in the respective clade. Phylogenetic data suggests a scenario in which an ancestral Met RTK might have evolved in a common ancestor of Bilateria. Met-LPs might have formed twice independently by similar tyrosine kinase domain acquisition events in lineages leading to Lophotrochozoa and Echinodermata (indicated by green plus signs). Loss of Met RTKs would have occurred in the lineage leading to Ecdysozoa (indicated by red minus signs). Protein domains: see legends of Figs 1, 3, and 4. Photo/image credits: *C*. *teleta*: François Michonneau (https://francoismichonneau.net/photos/capitella-telata) © 2018 by François Michonneau (CC-BY 4.0); *A*. *californica:* Genevieve Anderson*; C*. *elegans:* Bob Goldstein; *S*. *maritima*: Carlo Brena*; D*. *melanogaster*: Hans Smid; *A*. *planci*: Denis Zorzin; *B*. *belcheri*: Arthur Anker; *H*. *sapiens:* NASA.

Met and Met-LP RTKs are detected in the clades Lophotrochozoa and Echinodermata, and only Met RTKs are detected in the clade Chordata (Fig. 9). No Mets or Met-LPs are detectable in Ecdysozoa. The phylogenetic analysis data suggests a scenario wherein an acquisition of an intracellular tyrosine kinase domain by a plexin occurred in the lineage leading to Bilateria, generating an ancestral Met RTK from which all extant Met RTKs evolved (Fig. 9; gene 6 in Fig. S4B). Intriguingly, Met-LPs appear to have evolved twice independently by similar tyrosine kinase domain acquisition events in the lineages leading to Lophotrochozoa and Echinodermata. In this context, it is interesting to note that the Aplan Plexin-B1 and Met-LP1 genes (LOC110973988 and LOC110974068, respectively) lie next to each other on the same scaffold, suggesting that the diversification of Met-LPs in *A*. *planci* may have occurred through repeated duplication of a chromosomal segment containing a Plexin-B next to a Met-LP gene. Loss of Met genes may have occurred in the lineage leading to Ecdysozoa.

## Discussion

We have presented here a report on the evolution of plexin, semaphorin, and Met RTK proteins. Remarkably, plexins and semaphorins are already present in unicellular choanoflagellates, suggesting that these gene families have emerged in a common ancestor of choanoflagellates and Metazoa, more than 600 million years ago^35^. It has been proposed that choanoflagellates and Metazoa form together the clade Choanozoa^34^. As plexins and semaphorins are so far detected in all examined Choanozoa taxa, they may thus be considered a synapomorphy (feature unique to a clade) of Choanozoa.

The domain structure of plexins is largely conserved throughout all clades, suggesting that the receptor function of plexins is closely linked to their structural topology. This applies to both the extracellular parts of plexins, which form a ring structure^28,29^, and the intracellular part with its Ras-GAP domain^30^. In contrast to plexins, semaphorins display, -besides their obligatory N-terminal Sema domain-, highly diverse domain arrangements, which could indicate low topological constraint on how semaphorins are presented as ligands to plexin receptors.

Of note, several plexins and semaphorins of vertebrates carry C-terminal PDZ-binding motifs, which provide a binding site for PDZ-binding domains of proteins such as PDZ-RhoGEF, GIPC1, or PSD-95^53–55^. The plexins and semaphorins described in this study may also contain PDZ-binding motifs, but as PDZ-binding motifs are highly diverse in their sequences^56^, confident determination of PDZ binding will have to await experimental testing.

Interestingly, the comb jelly (Ctenophora) has the largest number of semaphorins among non-bilaterian Metazoa, and this expansion may be related to the development of a complex nervous system in Ctenophora^57^. The plexins and semaphorin gene families of Echinodermata and Vertebrata also underwent significant expansions that underlie the diversification of plexin/semaphorin functions in these organisms.

Previous studies of the genomes of unicellular Eukaryota and Metazoa have highlighted the early evolutionary appearance of cell adhesion machinery and related signaling pathways, including cadherins and integrins^35,36,38,43–45^. Our findings add to this concept in that plexins and semaphorins are already present in unicellular eukaryotes and may have been later adapted during evolution to regulate more complex intercellular interactions characteristic of Metazoa. Based on the structural similarity of the Sema domain with the beta-propeller domain of α integrins^19^, it could be speculated that the Sema domain evolved from a common ancestor that is shared with α integrins. Other components of plexins, such as the PSI, IPT, and Ras-GAP domains, are all present in several clades of unicellular eukaryotes (http://pfam.xfam.org/), suggesting that plexins may have formed in a common ancestor of choanoflagellates and Metazoa by domain shuffling of the evolutionary novel Sema domain with other existing domains.

What could be the original function of semaphorins and plexins? As the Ras-GAP activity of plexins seems to be directed towards small G-proteins of the Rap family, which are involved in spatio-temporal control of cell adhesion^58^, it is conceivable that plexins may act as negative regulators against positive cell adhesion forces from molecules such as cadherins and integrins. In this context, it is interesting to note that several choanoflagellate species can form facultative multicellular aggregates, e.g. rosettes^34^, and plexins may thus be involved in regulating the dynamics of these simple forms of multicellularity.

An intriguing discovery during our survey of Sema domain-containing proteins are the Met-LP RTKs, which are different from Met RTKs by harboring a structurally complete plexin-like extracellular domain. The absence of Met-LPs from the classic genetic model organisms worm, fly, mouse, and other vertebrates may be the reason that this type of RTK, to our knowledge, has not yet been described. The Met and Met-LP genes may be under different selection constraints regarding ligand binding: Mets have growth factors as ligands that are different from semaphorins; the ligands of Met-LPs are unknown, but it is conceivable that Met-LPs maintained semaphorins as ligands. This interesting possibility awaits further experimental testing.

In summary, the evolutionary data indicate that plexins and semaphorins predate the emergence of neurons in Metazoa, and that the axon guidance functions of plexins/semaphorins are therefore later specializations. The basic function of plexins and semaphorins may lie in balancing cell adhesion and cytoskeletal dynamics, thereby coordinating and adjusting cell-cell interactions and physical forces in unicellular and multicellular organisms.

## Methods

### Database survey for plexins, semaphorins, and Met-like RTKs

To identify plexins, semaphorins, and Sema domain-containing Met-like RTKs in unicellular eukaryotes and Metazoa, we surveyed genome databases by keyword (“Plexin”, “Semaphorin”, “Sema”) and BLASTP and TBLASTN searches (using a Sema domain peptide sequences as query). Searches were conducted at the NCBI Gene (www.ncbi.nlm.nih.gov/gene) and Protein (www.ncbi.nlm.nih.gov/protein) databases, Ensembl Genome database (www.ensembl.org), the Uniprot protein database (www.uniprot.org), the Mnemiopsis Genome Project Portal (research.nhgri.nih.gov/mnemiopsis), the JGI genome portals for *M*. *brevicollis* (genome.jgi.doe.gov/Monbr1) and *C*. *teleta* (genome.jgi.doe.gov/Capca1), and the PFAM protein domain database (pfam.xfam.org). For the ichtyosporean *C*. *fragrantissima* and the filasterean *C*. *owczarzaki*, we searched by TBLASTN the NCBI genome assemblies at www.ncbi.nlm.nih.gov/assembly/GCA_002024145.1 and www.ncbi.nlm.nih.gov/assembly/GCF_000151315.2, respectively.

The presence of a Sema domain was used as key criteria to designate a sequence as plexin, semaphorin, or Met RTK. If transcript data was available, gene models were inspected in genome browser view, and where applicable, protein sequences were edited to improve match to transcripts of homologous genes. Positions where predicted proteins are incomplete due to insufficient sequence coverage are indicated with poly-glycine stretches in protein sequence files and asterisks in cartoon models. Database URLs, source accession numbers, and editing notes for all protein sequences are listed in Table S1, and all the edited sequences are included as GenPept files (*.gp) in Supplemental data.

### Prediction of protein domains

Protein domain features were annotated with domain scan programs of NCBI CCD (www.ncbi.nlm.nih.gov/cdd), Expasy Prosite (prosite.expasy.org), and EBI HHMER (www.ebi.ac.uk/Tools/hmmer). Sema domains were further annotated by utilizing JPRED secondary structure prediction^59^ to identify beginning and end of the 7-blade beta-strand propeller pattern^19^ (see Fig. S2). Similarly, IPT domains were annotated by JPRED according to their secondary structure beta-strand pattern^27^. PSI domains were confirmed by their typical arrangement of up to 8 conserved cysteines^27^. Annotations were also refined by comparing sequences with HMM logos of domain conservation at the PFAM protein domain database (pfam.xfam.org).

Signal peptides were predicted with SignalP4.1 (www.cbs.dtu.dk/services/SignalP) and PHOBIUS (phobius.sbc.su.se) algorithms, and transmembrane domains with SOSUI (http://harrier.nagahama-i-bio.ac.jp/sosui) and TMHMM (www.cbs.dtu.dk/services/TMHMM) algorithms. Predicted protein domains are included in the GenPept files of Supplemental data as annotated features.

### Alignments and phylogenetic analysis

We performed alignments for phylogenetic analysis using the sequences of Sema domains and their adjacent PSI domains, which allowed construction of a tree including semaphorins, plexins, Mets, and Met-LPs. We aligned these sequences separately, using the algorithm M-Coffee of the T-Coffee software platform (tcoffee.crg.cat), which combines different alignment algorithms to produce an optimized alignment^60^. We combined the aligned sequences with the aid of the Fasta alignment joiner (users-birc.au.dk/biopv/php/fabox/alignment_joiner.php#). Phylogenetic trees were inferred with MrBayes software^61,62^, taking in account two partitions (Sema and PSI domains). The program was set to find the best model of substitution during the analysis and found that the WAG model is the one that better describes the evolution of the proteins under study. Since the computational task was long, we used the CIPRES Portal (www.phylo.org)^63^ for the maximum time allowed (168 hours of eight servers) to complete the task. We obtained the consensus tree after 15 million steps of a Markov chain into two runs of four chains each (with 25% of burning). We examined the parameters convergence using Tracer software (github.com/beast-dev/tracer/releases/tag/v1.7.1) and visualized the tree using the FigTree software (tree.bio.ed.ac.uk/software/figtree). The infile containing the alignment and the script used here are included as supplemental data files.

### Gene duplication analysis

To develop a hypothesis on the gene duplication events that gave rise to the extant plexins and semaphorins, we drew the most accepted topology of the taxa under study^34^ and mapped the sequences found in each genome into this representation (Fig. S4A for plexins, Fig. S5A for semaphorins). The next step was to follow the phylogenetic hypotheses (Figs 6 and 7) and infer the minimal number of gene duplication events required to explain the copies found in each taxon. For that, the gene at the base of the tree (the ancestral gene of choanoflagellate and metazoan plexins or semaphorins, respectively) was assigned as gene 1 (in red). We maintained the gene 1 in the branches until finding a duplication within one of the taxa. Therefore, all red branches of the tree are here considered descendants of gene 1. For example, in the plexin phylogenetic tree (see Fig. S4B), we observe, after the split of choanoflagellate plexins the semaphorin clade, and after that, we have the first clade of non-bilaterian plexins. In this clade, we found a polytomy, with one Porifera sequence (Aquee Plexin-1), one Ctenophora sequence (Mleid Plexin-1) and five other Porifera plexins (Aquee Plexin-A1-A5). Thus, we assigned a duplication that took place after the divergence of Porifera and termed the gene formed after gene 1 duplication as gene 2. Thus, those sequences that branch off more basally in the tree, such as Mleid Plexin-1 and Aquee Plexin-1, were considered descendants of gene 1 (branches in red in Fig. S4B), and the remaining five Aquee sequences (Plexin-A1-A5) descendants of gene 2. Following the tree, we assigned genes 3, 4 and 5 accordingly. Gene 3 is a duplication of gene 1 that occurred after the divergence of Ctenophora, while genes 4 and 5 duplicated from gene 1 after the split of Metazoa and Choanoflagellidae. Ctenophora inherited genes 4 and 5, while Placozoa have only gene 4 and Cnidaria only gene 5. To assign gene duplication events necessary to explain the current copies found in each taxon, we followed these rules: the gene number order follows the phylogenetic tree from base to tips. If a plexin/semaphorin is within a monophyletic group (see for example gene 6 descendants in Fig. S4), all of their descendants are assigned to this gene, except if the group comprises two copies of the same taxon. If the copies of the same taxon form a monophyletic clade, they are assigned to the same gene number (for example gene 2 descendants of Fig. S4B). If the copies do not form monophyletic clades, new gene duplications are assigned (for example genes 7 and 8 of Fig. S4).

### Protein conservation plot

A conservation plot of plexin sequences was generated with the plotcon algorithm (emboss.bioinformatics.nl/cgi-bin/emboss/plotcon).

## Supplementary information

Supplementary Material

Table S1

File S1

File S2

File S3

## Data Availability

All data generated in this study are included in this published article and its Supplemental Data files.
