# Supplementary material for: Origin and evolution of plexins, semaphorins, and Met receptor tyrosine kinases
Source: Sci Rep. 2019 Feb 13;9:1970. doi: 10.1038/s41598-019-38512-y (PMC6374515; doi:10.1038/s41598-019-38512-y)
Supplement: Supplementary file 1 — Supplementary Material [file 41598_2019_38512_MOESM1_ESM.pdf]

SUPPLEMENTARY MATERIAL

Origin and evolution of plexins, semaphorins, and Met receptor tyrosine kinases

Chrystian Junqueira Alves, Karla Yotoko, Hongyan Zou, Roland H. Friedel

Supplemental data files

Table S1 protein source accession numbers.xlsx

List of plexin, semaphorin, Met, and Met-LP sequences compiled for this study, including source databases, accession numbers, and editing notes.

File S1 sequences.zip

ZIP file containing sequence files of all proteins that were compiled for this study in GenPept format (*.gp). Sequence files also contain information about annotated domain features.

File S2 infile.zip

ZIP file of Nexus alignment file (.nex file type) of Sema plus PSI domain of all Sema domain containing proteins.

File S3 all_complete.zip

ZIP file of phylogenetic tree of all Sema domain containing proteins (.tre file type). Source file for the tree representations shown in Fig. 5-7.

Supplemental figure legends

Figure S1. Alignment of human and choanoflagellate plexins

Alignment of human and choanoflagellate plexins was calculated using MUSCLE (www.ebi.ac.uk/Tools/msa/muscle; default settings) and visualized using Jalview (www.jalview.org). Predicted protein domains are indicated below the alignment.

Hsapi, *H. sapiens*; Mbrev, *M. brevicollis*; Srose, *S. rosetta*.

Figure S2. Secondary structure prediction of Sema domain of choanoflagellate plexin

JPRED secondary structure prediction of Sema domain of Plexin-1 of choanoflagellate *M. brevicollis*. Green arrows predict beta-strands, red boxes alpha-helices. The seven blades of the Sema domain can be recognized by the beta-strand pattern. The first beta-strand, marked as (7), is part of blade 7. Two extrusions, between blade 1 and 2 and inside blade 5, are recognizable by the presence of alpha-helices.

Figure S3. Alignments of Sema domains of choanoflagellate and human plexins and semaphorins

Alignments of human and choanoflagellate Sema plus PSI domains of plexins (A) and semaphorins (B) were created with MUSCLE. Colored arrows indicate predicted beta-strands of the seven blades of the Sema domain; gray boxes indicate extrusions 1 and 2. Conservation track indicates: "*", fully conserved amino acids (aa); ":", conservation of aa with strongly similar properties; ".", conservation of aa with weakly similar properties. Conserved cysteines of the Sema domains are highlighted in yellow (Cys1-8). Further annotations are derived from structural data of mouse Sema6A and Plexin-A2 (Nogi et al., 2010). Amino acids that are predicted to take part in Plexin-A / Sema6 binding are bolded and also indicated by black dots.

Figure S4. Gene duplication model based on plexin phylogenetic tree

A) Representation of the most accepted phylogenetic tree of the taxa under study. Below each taxon, the plexin, Met, and Met-LP sequences found in each genome are indicated. Those sequences contained in monophyletic clades in a taxon were represented as only one gene group. We numbered the genes from the base to the tips of the gene phylogeny (see Fig. 6) and searched for the minimum number of duplications sufficient to explain the number of sequences found in each taxon. TK, tyrosine kinase domain.

B) Topology of the phylogenetic hypothesis inferred for plexins, Mets, and Met-LPs (related to Fig. 6) with numbers of hypothetical ancestral genes indicated at internal nodes of the tree. The red branches indicate the sequences assigned as descendants of gene 1, which originated in an ancestor of Choanoflagellida and Metazoa. The green branches indicate sequences assigned as descendants of genes 2 - 5, which originated in non-bilaterian organisms. Purple branches indicate sequences assigned to genes 6 – 10 that originated in Protostomia. The blue branches indicate the sequences assigned to gene 11 that originated in Deuterostomia.

Figure S5. Gene duplication model based on semaphorin phylogenetic tree

A) Representation of the most accepted phylogenetic tree of the taxa under study. Below each taxon, the semaphorins found in each genome are indicated. Those sequences contained in a monophyletic clade in a taxon were represented as only one gene group. We numbered the genes from the base to the tips of the gene phylogeny (see Fig. 7) and searched for the minimum number of duplications that are sufficient to explain the number of sequences found in each taxon. FN, fibronectin type III domain; TH, thrombospondin type 1 domain; IG, immunoglobulin-like domain.

B) Topology of the phylogenetic hypothesis inferred for semaphorin sequences (related to Fig. 7) with the gene numbers indicated at internal nodes of the tree. The red branches indicate the sequences assigned as descendants from gene 1, which originated in an ancestor of Choanoflagellida and Metazoa. The green branches indicate sequences assigned to genes 2 - 6, which originated in non-bilaterian organisms. The purple branches indicate sequences assigned to genes 7 and 8 that originated in Protostomia. The blue branches indicate the sequences assigned to genes 9, 10, and 11 that originated in Deuterostomia.

Fig. S1 Alignment of human and choanoflagellate plexins

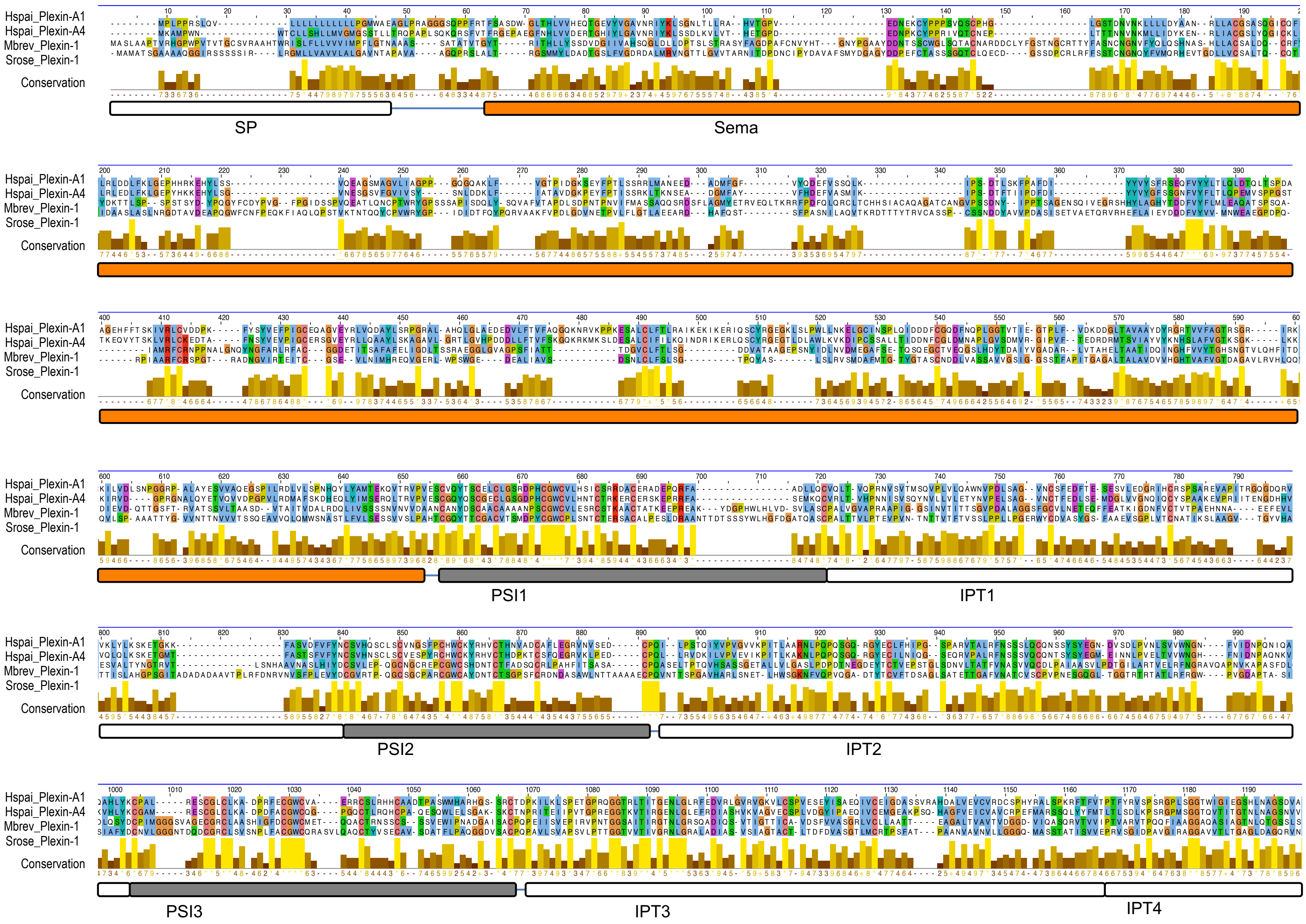

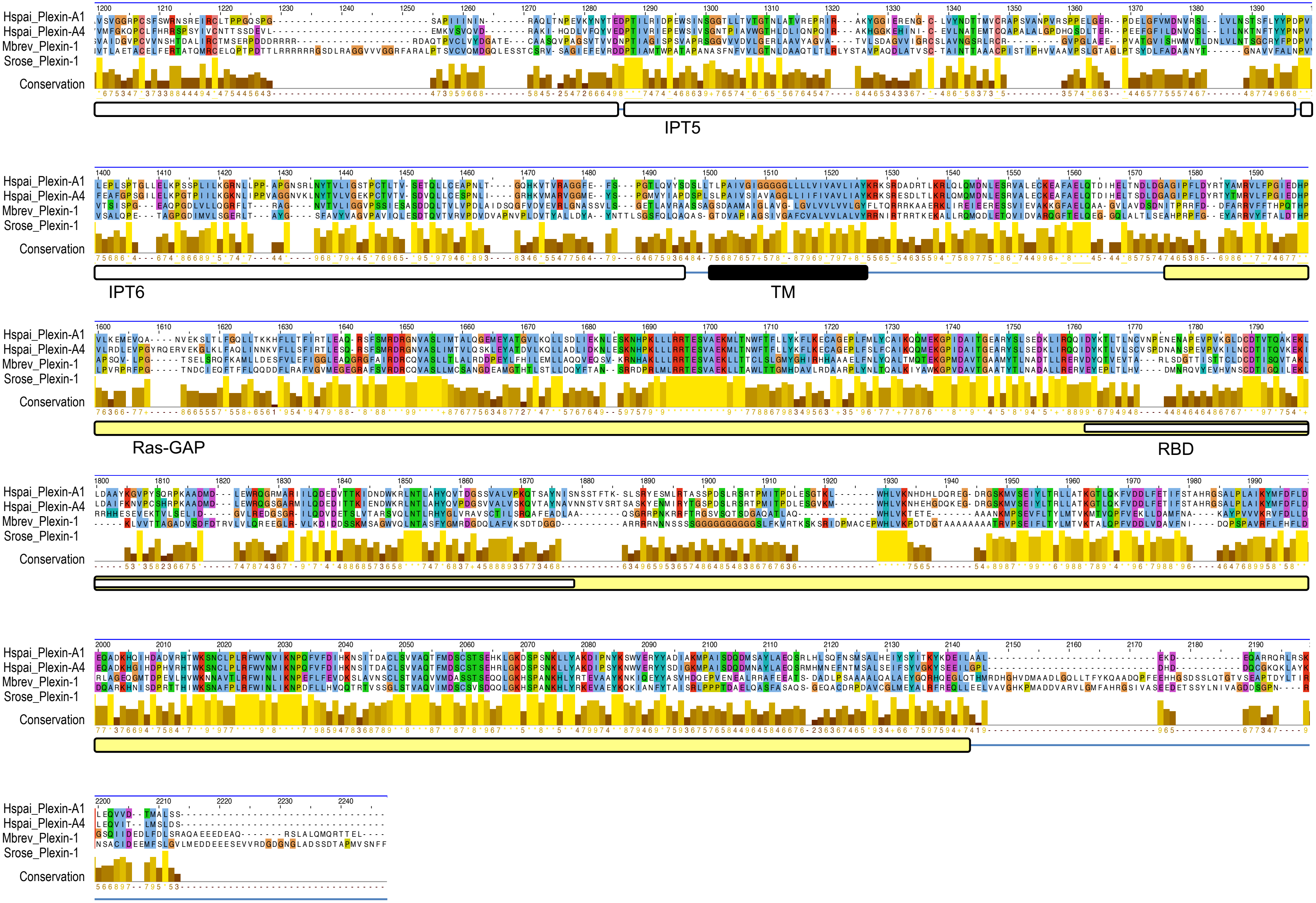

Hspai_Plexin-A1
Hspai_Plexin-A4
Mbrev_Plexin-1
Srose_Plexin-1
Conservation
IPT5
IPT6
TM
Ras-GAP
RBD

Fig. S2 Secondary structure prediction of Sema domain of choanoflagellate plexin

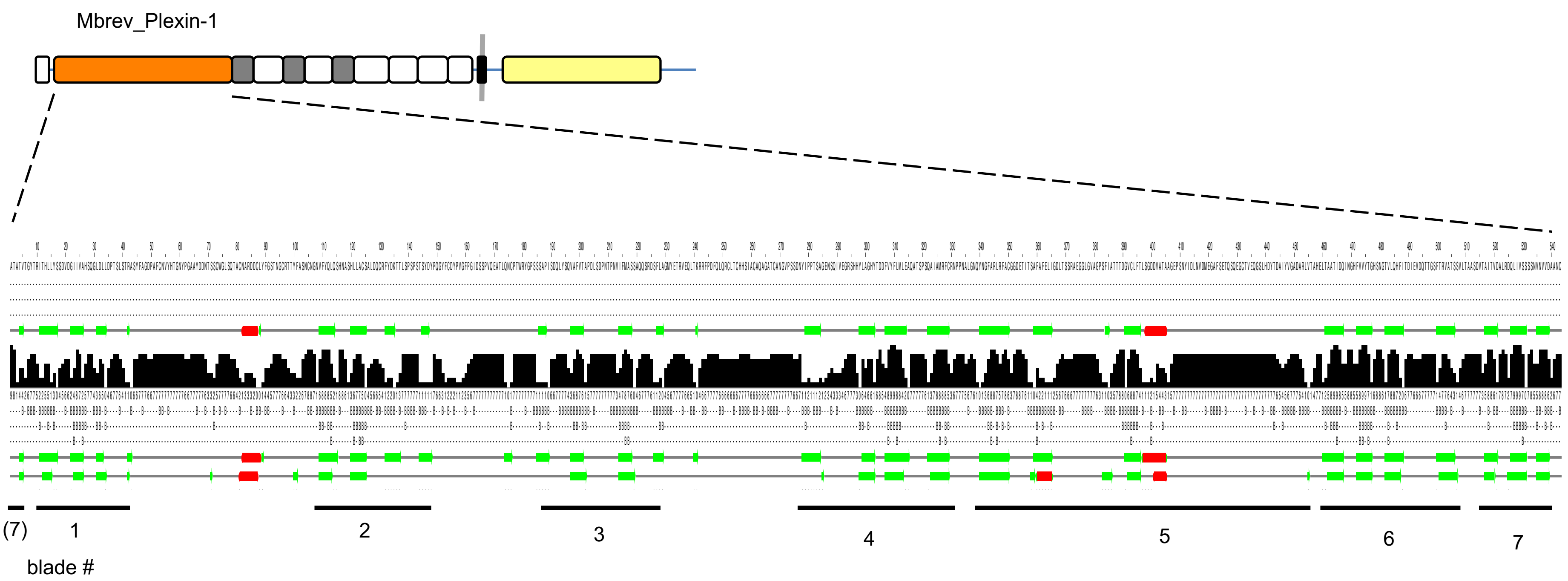

JPRED 4
http://www.compbio.dundee.ac.uk/jpred/

Fig. S3 Alignments of Sema domains of choanoflagellate and human plexins and semaphorins

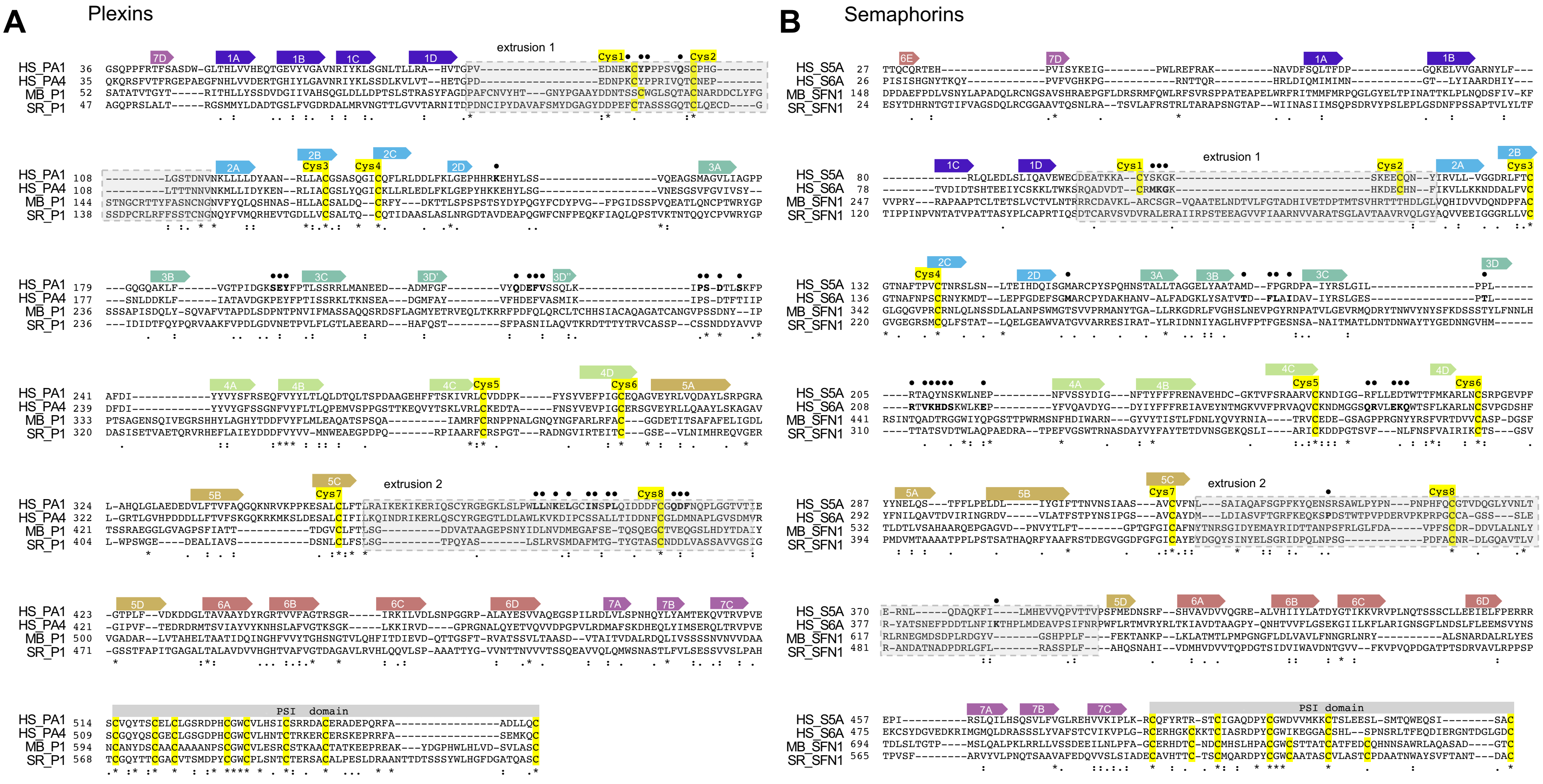

HS *Homo sapiens*
MB *Monosiga brevicollis*
SR *Salpingoeca rosetta*

PA1 Plexin-A1
PA4 Plexin-A4
P1 Plexin-1

S5A Sema5A
S6A Sema6A
SFN1 Sema-FN1

Fig. S4 A Model of major gene duplication events in evolution of plexins and Met RTKs

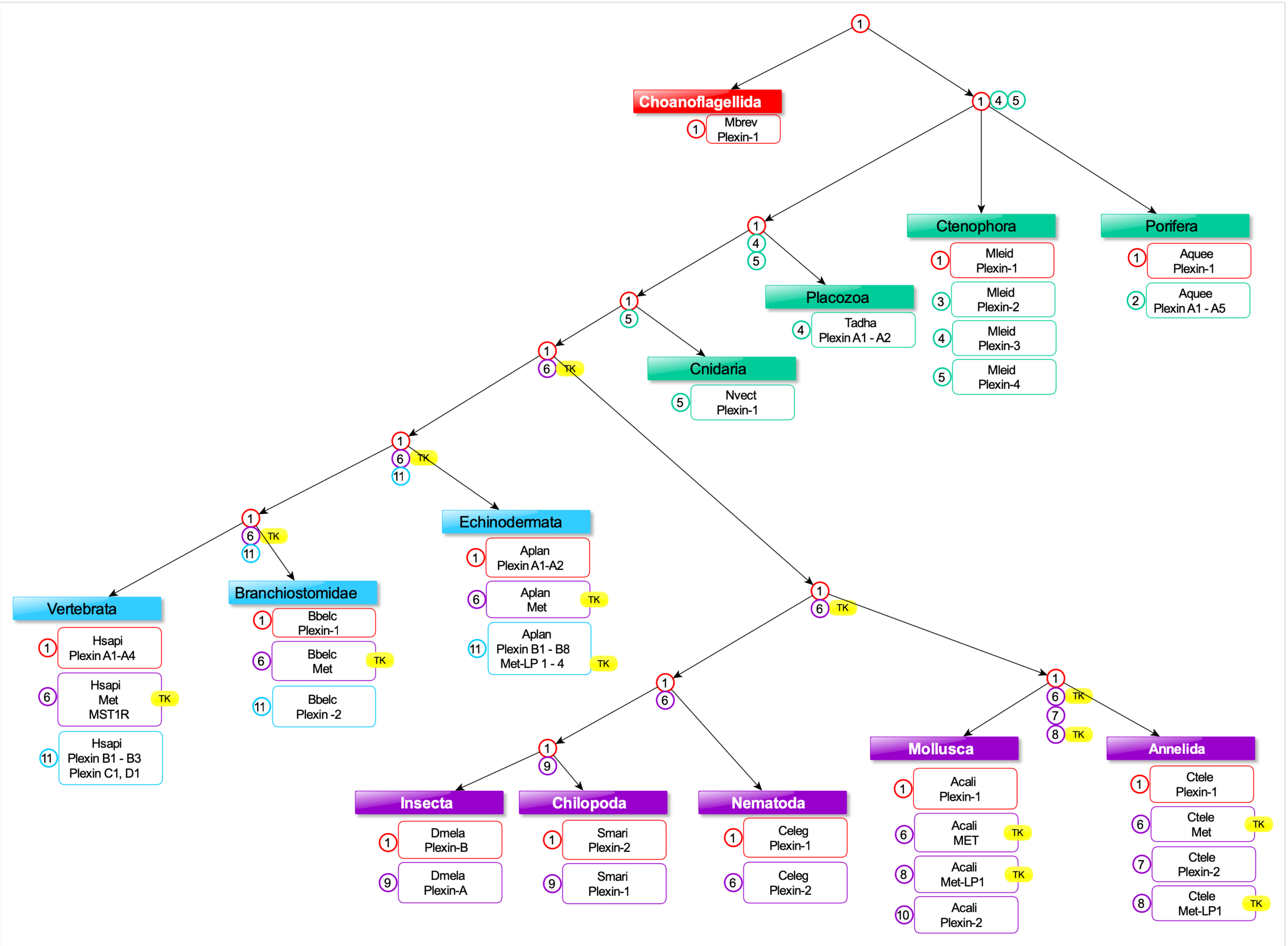

Fig. S4 B

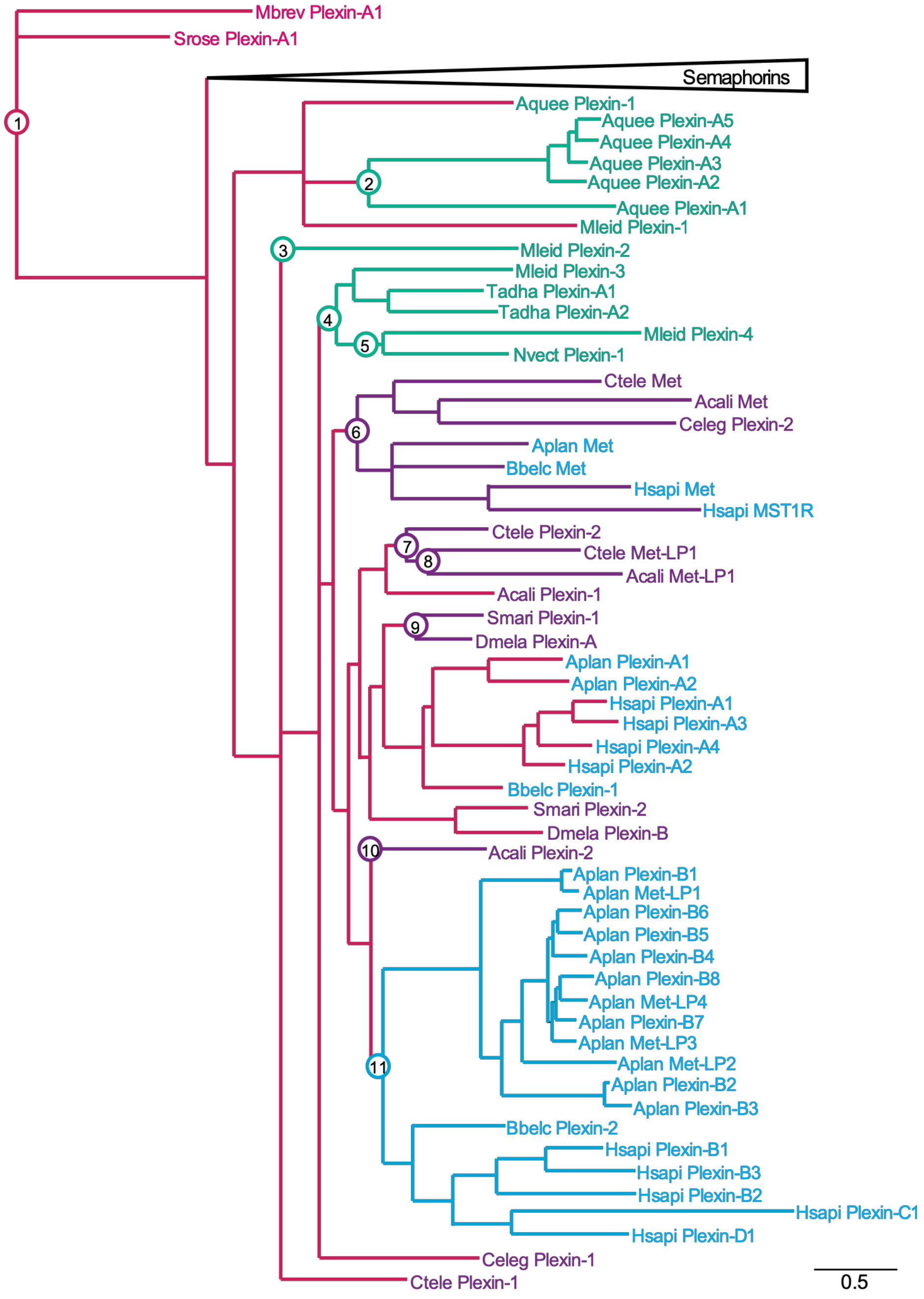
Mbrev Plexin-A1
Srose Plexin-A1
Semaphorins
Aquee Plexin-1
Aquee Plexin-A5
Aquee Plexin-A4
Aquee Plexin-A3
Aquee Plexin-A2
Aquee Plexin-A1
Mleid Plexin-1
Mleid Plexin-2
Mleid Plexin-3
Tadha Plexin-A1
Tadha Plexin-A2
Mleid Plexin-4
Nvect Plexin-1
Ctele Met
Acali Met
Celeg Plexin-2
Aplan Met
Bbelc Met
Hsapi Met
Hsapi MST1R
Ctele Plexin-2
Ctele Met-LP1
Acali Met-LP1
Acali Plexin-1
Smari Plexin-1
Dmela Plexin-A
Aplan Plexin-A1
Aplan Plexin-A2
Hsapi Plexin-A1
Hsapi Plexin-A3
Hsapi Plexin-A4
Hsapi Plexin-A2
Bbelc Plexin-1
Smari Plexin-2
Dmela Plexin-B
Acali Plexin-2
Aplan Plexin-B1
Aplan Met-LP1
Aplan Plexin-B6
Aplan Plexin-B5
Aplan Plexin-B4
Aplan Plexin-B8
Aplan Met-LP4
Aplan Plexin-B7
Aplan Met-LP3
Aplan Met-LP2
Aplan Plexin-B2
Aplan Plexin-B3
Bbelc Plexin-2
Hsapi Plexin-B1
Hsapi Plexin-B3
Hsapi Plexin-B2
Hsapi Plexin-C1
Hsapi Plexin-D1
Celeg Plexin-1
Ctele Plexin-1
1
2
3
4
5
6
7
8
9
10
11
0.5

Fig. S5 A Model of major gene duplication events in evolution of semaphorins

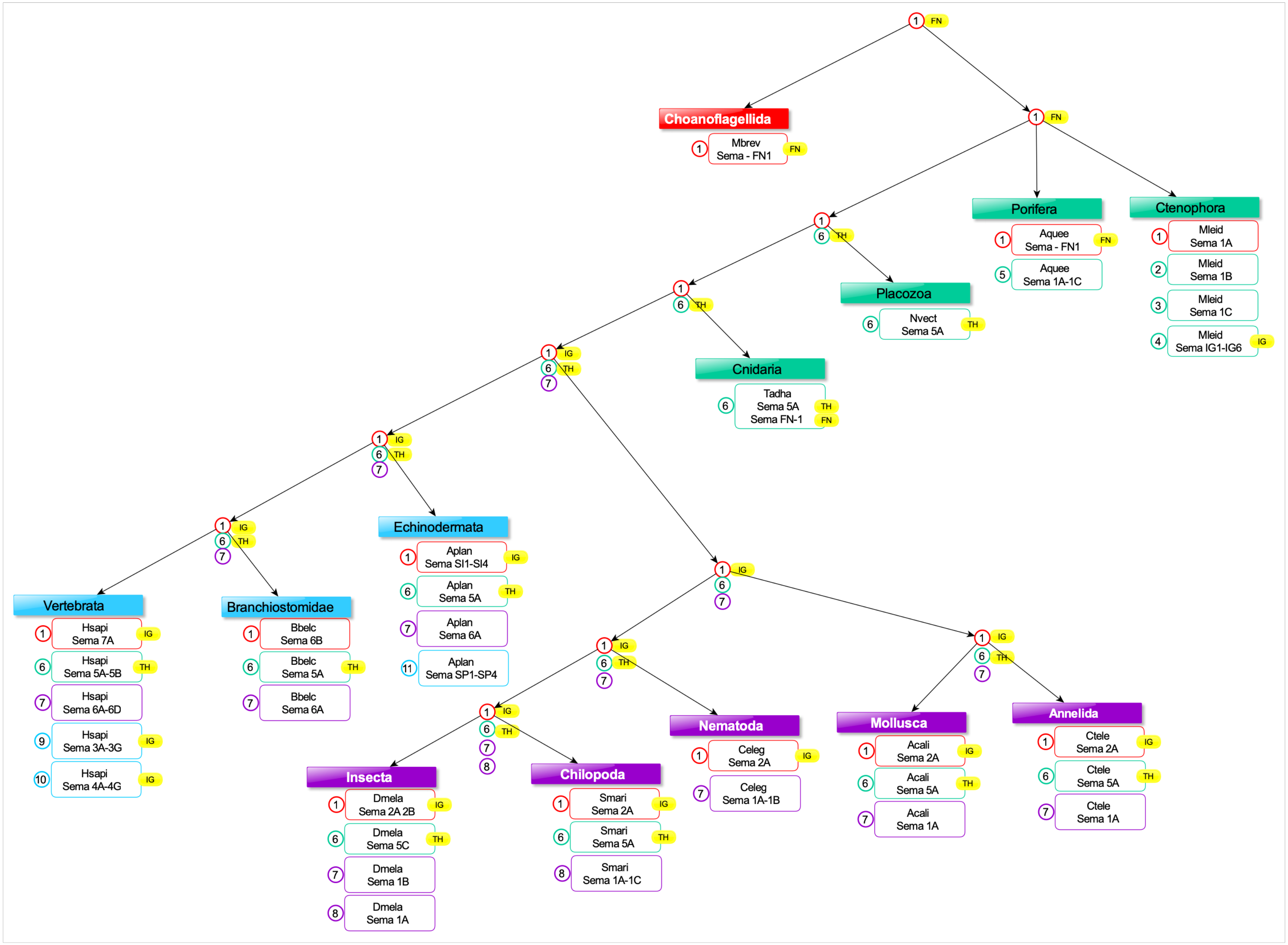

Fig. S5 B

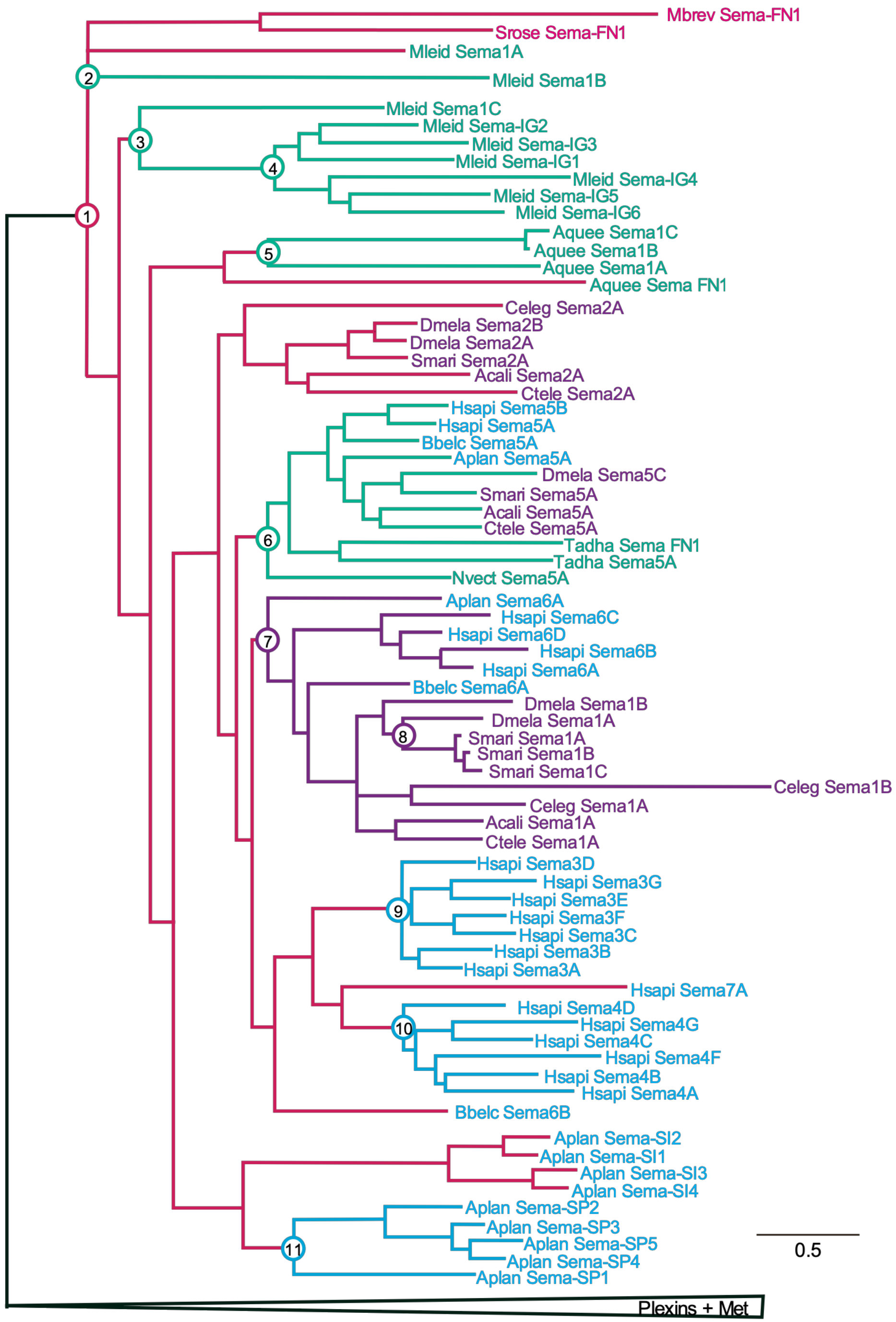

Mbrev Sema-FN1
Srose Sema-FN1
Mleid Sema1A
Mleid Sema1B
Mleid Sema1C
Mleid Sema-IG2
Mleid Sema-IG3
Mleid Sema-IG1
Mleid Sema-IG4
Mleid Sema-IG5
Mleid Sema-IG6
Aquee Sema1C
Aquee Sema1B
Aquee Sema1A
Aquee Sema FN1
Celeg Sema2A
Dmela Sema2B
Dmela Sema2A
Smari Sema2A
Acali Sema2A
Ctele Sema2A
Hsapi Sema5B
Hsapi Sema5A
Bbelc Sema5A
Aplan Sema5A
Dmela Sema5C
Smari Sema5A
Acali Sema5A
Ctele Sema5A
Tadha Sema FN1
Tadha Sema5A
Nvect Sema5A
Aplan Sema6A
Hsapi Sema6C
Hsapi Sema6D
Hsapi Sema6B
Hsapi Sema6A
Bbelc Sema6A
Dmela Sema1B
Dmela Sema1A
Smari Sema1A
Smari Sema1B
Smari Sema1C
Celeg Sema1B
Celeg Sema1A
Acali Sema1A
Ctele Sema1A
Hsapi Sema3D
Hsapi Sema3G
Hsapi Sema3E
Hsapi Sema3F
Hsapi Sema3C
Hsapi Sema3B
Hsapi Sema3A
Hsapi Sema7A
Hsapi Sema4D
Hsapi Sema4G
Hsapi Sema4C
Hsapi Sema4F
Hsapi Sema4B
Hsapi Sema4A
Bbelc Sema6B
Aplan Sema-SI2
Aplan Sema-SI1
Aplan Sema-SI3
Aplan Sema-SI4
Aplan Sema-SP2
Aplan Sema-SP3
Aplan Sema-SP5
Aplan Sema-SP4
Aplan Sema-SP1
Plexins + Met
1
2
3
4
5
6
7
8
9
10
11
0.5
